# A mini-TGA protein modulates gene expression through heterogeneous association with transcription factors

**DOI:** 10.1093/plphys/kiac579

**Published:** 2022-12-15

**Authors:** Špela Tomaž, Marko Petek, Tjaša Lukan, Karmen Pogačar, Katja Stare, Erica Teixeira Prates, Daniel A Jacobson, Jan Zrimec, Gregor Bajc, Matej Butala, Maruša Pompe Novak, Quentin Dudley, Nicola Patron, Ajda Taler-Verčič, Aleksandra Usenik, Dušan Turk, Salomé Prat, Anna Coll, Kristina Gruden

**Affiliations:** Department of Biotechnology and Systems Biology, National Institute of Biology, 1000 Ljubljana, Slovenia; Jožef Stefan International Postgraduate School, 1000 Ljubljana, Slovenia; Department of Biotechnology and Systems Biology, National Institute of Biology, 1000 Ljubljana, Slovenia; Department of Biotechnology and Systems Biology, National Institute of Biology, 1000 Ljubljana, Slovenia; Department of Biotechnology and Systems Biology, National Institute of Biology, 1000 Ljubljana, Slovenia; Department of Biotechnology and Systems Biology, National Institute of Biology, 1000 Ljubljana, Slovenia; Biosciences Division, Oak Ridge National Laboratory,, Oak Ridge, Tennessee 37831, USA; Biosciences Division, Oak Ridge National Laboratory,, Oak Ridge, Tennessee 37831, USA; Department of Biotechnology and Systems Biology, National Institute of Biology, 1000 Ljubljana, Slovenia; Department of Biology, Biotechnical Faculty, University of Ljubljana, 1000 Ljubljana, Slovenia; Department of Biology, Biotechnical Faculty, University of Ljubljana, 1000 Ljubljana, Slovenia; Department of Biotechnology and Systems Biology, National Institute of Biology, 1000 Ljubljana, Slovenia; School for Viticulture and Enology, University of Nova Gorica, 5271 Vipava, Slovenia; Earlham Institute, Norwich Research Park, Norwich NR4 7UZ, UK; Earlham Institute, Norwich Research Park, Norwich NR4 7UZ, UK; Department of Biochemistry and Molecular and Structural Biology, Jožef Stefan Institute, 1000 Ljubljana, Slovenia; Faculty of Medicine, Institute of Biochemistry and Molecular Genetics, University of Ljubljana, 1000 Ljubljana, Slovenia; Department of Biochemistry and Molecular and Structural Biology, Jožef Stefan Institute, 1000 Ljubljana, Slovenia; Centre of Excellence for Integrated Approaches in Chemistry and Biology of Proteins, 1000 Ljubljana, Slovenia; Department of Biochemistry and Molecular and Structural Biology, Jožef Stefan Institute, 1000 Ljubljana, Slovenia; Centre of Excellence for Integrated Approaches in Chemistry and Biology of Proteins, 1000 Ljubljana, Slovenia; Department of Plant Development and Signal Transduction, Centre for Research in Agricultural Genomics, 08193 Cerdanyola, Barcelona, Spain; Department of Biotechnology and Systems Biology, National Institute of Biology, 1000 Ljubljana, Slovenia; Department of Biotechnology and Systems Biology, National Institute of Biology, 1000 Ljubljana, Slovenia

## Abstract

TGA (TGACG-binding) transcription factors, which bind their target DNA through a conserved basic region leucine zipper (bZIP) domain, are vital regulators of gene expression in salicylic acid (SA)-mediated plant immunity. Here, we investigated the role of StTGA2.1, a potato (*Solanum tuberosum*) TGA lacking the full bZIP, which we named a mini-TGA. Such truncated proteins have been widely assigned as loss-of-function mutants. We, however, confirmed that *StTGA2.1* overexpression compensates for SA-deficiency, indicating a distinct mechanism of action compared with model plant species. To understand the underlying mechanisms, we showed that StTGA2.1 can physically interact with StTGA2.2 and StTGA2.3, while its interaction with DNA was not detected. We investigated the changes in transcriptional regulation due to *StTGA2.1* overexpression, identifying direct and indirect target genes. Using *in planta* transactivation assays, we confirmed that StTGA2.1 interacts with StTGA2.3 to activate *StPRX07*, a member of class III peroxidases (StPRX), which are known to play role in immune response. Finally, via structural modeling and molecular dynamics simulations, we hypothesized that the compact molecular architecture of StTGA2.1 distorts DNA conformation upon heterodimer binding to enable transcriptional activation. This study demonstrates how protein truncation can lead to distinct functions and that such events should be studied carefully in other protein families.

## Introduction

Plants have developed efficient strategies to withstand the invasion of surrounding microbes. Pathogen recognition is mediated by plant cell-surface and intracellular receptors, triggering a cascade of intracellular reactions, orchestrated by phytohormones, ultimately leading to a finely modulated transcriptional reprogramming (Zhou and Zhang, 2020). Regulation of defense-related gene expression is among the most fundamental aspects of the immune response, involving multiple transcription factors and cofactor proteins. Since their initial discovery in tobacco (*Nicotiana tabacum*) over 30 years ago (Katagiri et al., 1989), the importance of TGA (TGACG-binding) transcription factors in plant immunity, as well as modulation of other cellular processes, has been widely studied (Gatz, 2013).

TGAs are a group of transcription factors belonging to the basic region leucine zipper (bZIP) protein family. Their mechanism of action has been thoroughly studied in Arabidopsis (*Arabidopsis thaliana*), where the 10 Arabidopsis TGAs (AtTGAs) group into five clades (Jakoby et al., 2002). Clade II members, AtTGA2, AtTGA5, and AtTGA6, are essential regulators of the salicylic acid (SA)-mediated defense response, where they play a redundant, yet vital role in establishing resistance following infection (Zhang et al., 2003; Zhou and Zhang, 2020). They coregulate the expression of key defense-related genes and genes involved in SA synthesis through interaction with NON-EXPRESSOR OF PR (NPR) cofactors (Zhang et al., 1999; Ding et al., 2018), while also participating in jasmonic acid and ethylene-mediated signaling (Zander et al., 2010). Structurally, TGAs consist of an intrinsically disordered N terminus of varying length, a conserved bZIP domain, which entails a basic region and a leucine zipper, and a C-terminal region that contains a putative Delay of Germination 1 (DOG1) domain (Tomaž et al., 2022). TGAs bind their target DNA through the bZIP basic region, while the leucine zipper is important for protein dimerization (Thurow et al., 2005) and oligomerization (Boyle et al., 2009). The TGACG core sequence is sufficient for TGA binding, although high-throughput DNA-binding studies revealed the TGACGTCA palindrome as the representative binding motif (Thibaud-Nissen et al., 2006; O’Malley et al., 2016).

The molecular mechanisms of TGA-mediated regulation involve complex interactions between TGAs and other proteins (Gatz, 2013). For example, the SA-receptor NPR1 interacts with AtTGA2 to activate the expression of the *PATHOGENESIS-RELATED-1* (*PR-1*) gene expression (Zhang et al., 1999; Backer et al., 2019), but the mechanistic aspect of this cooperation is not yet entirely clear. Several studies suggest that AtTGA2 acts as a constitutive repressor of *PR-1* in absence of biotic stress (Zhang et al., 2003; Rochon et al., 2006; Kesarwani et al., 2007). Its repressive activity is then alleviated through NPR1 interaction with AtTGA2 N terminus, affecting the binding stoichiometry and leading to the formation of a transcriptional activation complex (Rochon et al., 2006; Boyle et al., 2009). Additionally, other reports propose NPR1 interacts with TGAs not yet bound to DNA and indicate it could facilitate TGA binding to target promoter (Johnson et al., 2008). Furthermore, regulatory proteins, such as WRKY50 (Hussain et al., 2018) and histone acyltransferase (HAC) transcription factors (Jin et al., 2018), have also been shown to contribute to AtTGA2 transcriptional function.

Although the results obtained in Arabidopsis provide a molecular framework for understanding the role of TGAs in plant immunity, we know much less about their function in crops. The involvement of TGAs in biotic stress response has been reported in several species, including rice (*Oryza sativa*) (Moon et al., 2018), soybean (*Glycine max*) (Lawaju et al., 2018), strawberry (*Fragaria* × *ananassa*) (Feng et al., 2020), tobacco (Thurow et al., 2005), and tomato (*Solanum lycopersicum*) (Ekengren et al., 2003). Potato (*Solanum tuberosum*) is one of the most widely grown crops (FAO, 2020) and tuber production is severely threatened by pathogen infections. Several transcription factor families have been associated with the regulation of potato defense response (Chacón-Cerdas et al., 2020), but the mechanisms underlying potato TGA (StTGA) activity remain largely unexplored.

Here, we identify the mini-TGA StTGA2.1, a potato clade II TGA, which lacks most of the bZIP DNA-binding domain and has a shorter N terminus. We hypothesize that StTGA2.1 cannot bind DNA by itself because of the truncated bZIP and therefore modulates gene expression through its interaction with additional DNA-binding StTGAs. By combining *in vivo* and *in vitro* functional studies, we confirm the role of StTGA2.1 in potato immunity. Furthermore, using *in silico* structural analysis and molecular dynamics (MD) simulations, we provide insights into the molecular basis for a different mechanism of action of StTGA2.1 compared to other StTGAs.

## Results

### Potato encodes clade II TGAs with truncated bZIP domain

In previous work, we investigated gene expression in response to viral infection in nontransgenic-resistant potato (NT) and its transgenic derivative (NahG), which is impaired in SA accumulation and thus sensitive to infection (Baebler et al., 2014). To identify the TGA transcription factors involved in potato immunity, we examined the expression patterns of the 14 StTGA genes, orthologs of AtTGAs (Supplemental Tables S1 and S2). Notably, *Sotub10g022560* was up-regulated in infected NahG transgenic plants, but not in the parental lines, suggesting that it may be an important component of SA signaling.

**Figure 1:**
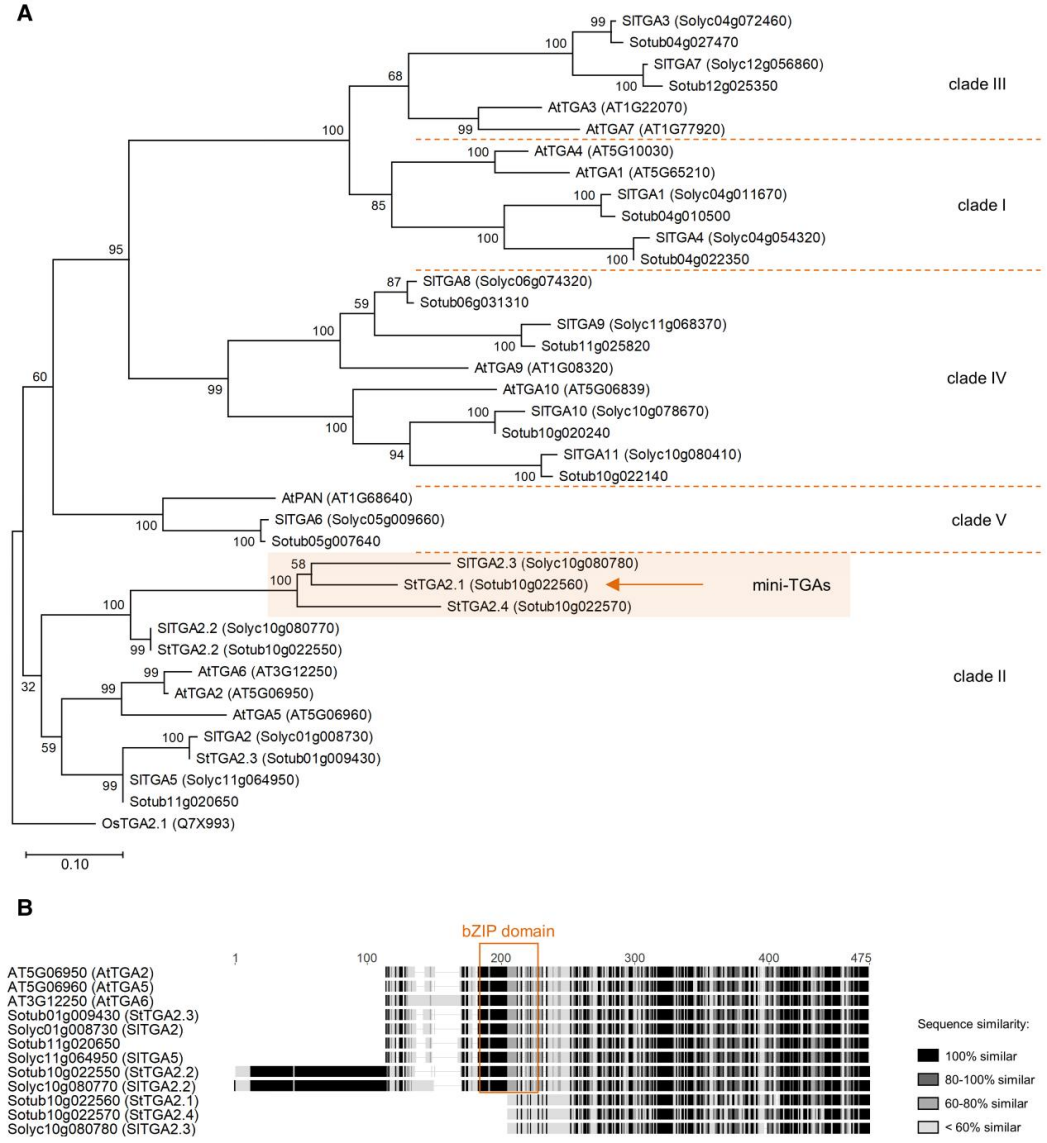
Phylogenetic analysis and domain characterization of StTGAs. A, A rooted phylogenetic tree of potato, tomato, and Arabidopsis TGAs. The mini-TGA branch is shaded and StTGA2.1 is marked (arrow). The branch length scale represents the number of amino acid substitutions per site. The rice OsTGA2.1 (Chern et al., 2001) serves as tree root. B, Protein sequence alignment of clade II TGAs, showing the position of the bZIP domain (box) and the shorter sequences of mini-TGA members, StTGA2.1, StTGA2.4, and SlTGA2.3. The alignment is colored with the Geneious Prime (https://www.geneious.com/) sequence similarity color scheme, based on the identity score matrix. Sequence numbering (aa) is shown above the alignment.

To classify the StTGAs, we conducted a phylogenetic analysis of all candidate potato proteins, along with the 10 AtTGAs and 13 TGAs from tomato (SlTGAs) (Hou et al., 2019; Lemaire-Chamley et al., 2022). Interestingly, the three clade II AtTGAs are orthologous to five StTGAs and four SlTGAs (Figure 1A). Three closely related members of this clade, including *Sotub10g022560*, named StTGA2.1, StTGA2.4 (*Sotub10g022570*), and SlTGA2.3 (*Solyc10g080780*) (Hou et al., 2019; Lemaire-Chamley et al., 2022), have shorter protein sequences than other TGAs (Figure 1B). Domain prediction studies showed that they retain the putative C-terminal DOG1 domain; however, the bZIP domain is almost completely lost, retaining only a partial zipper region. In addition, their N terminus is very short and dissimilar to the N termini of other clade II TGAs. We named these three proteins mini-TGAs.

By targeted sequencing of a ∼36.5 kb region on chromosome 10, where *StTGA2.1*, *StTGA2.2* (*Sotub10g022550*), and *StTGA2.4* loci are colocated, we confirmed the reduced length of *StTGA2.1* and *StTGA2.4* in a tetraploid cultivar that was used for further analyses (Supplemental Figure S1).

### StTGA2.1 improves immune response in SA-deficient potato

**Figure 2:**
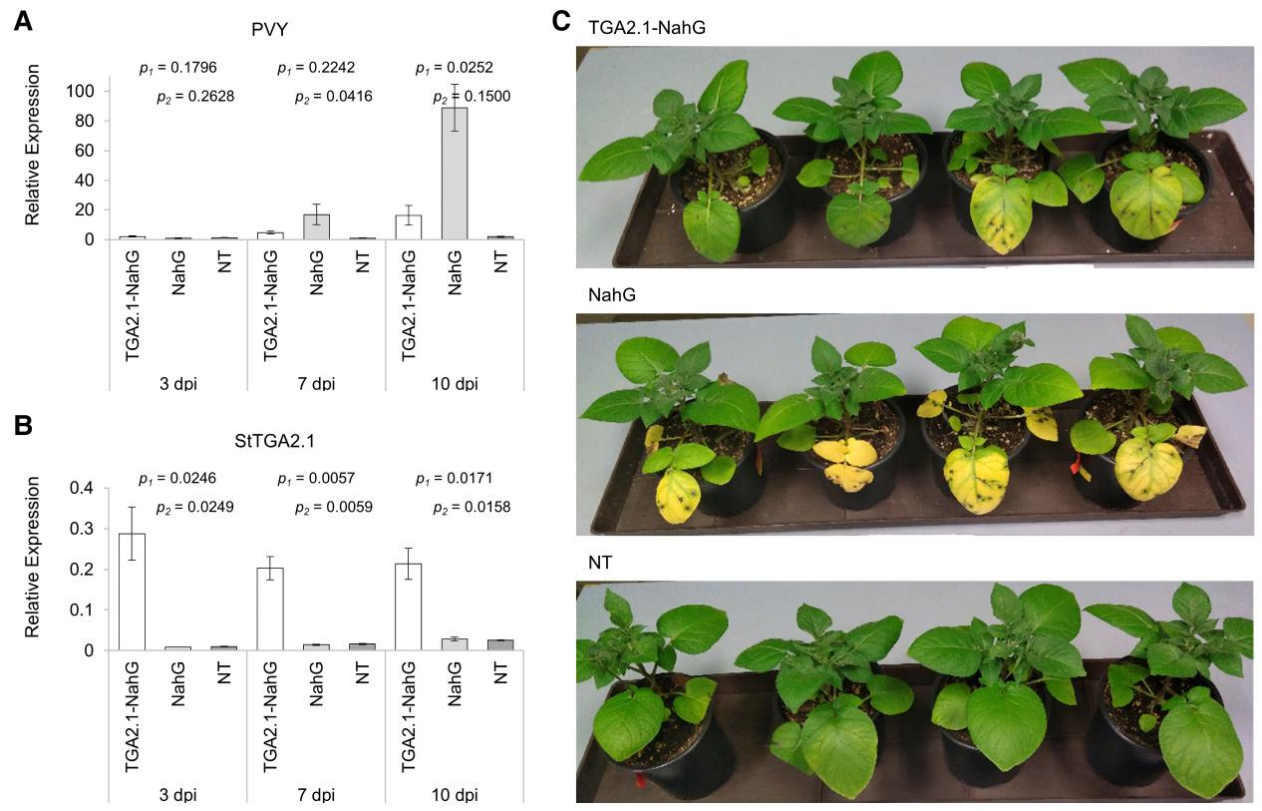
StTGA2.1 attenuates PVY replication in SA-deficient plants. Relative expression levels of (A) PVY and (B) *StTGA2.1* in PVY-infected leaves of DEX-treated TGA2.1-NahG line 12 (white), NahG (light grey), and NT (dark grey) plants at 3, 7, and 10 dpi. Average values ± SE from three biological replicates are shown. Significance (*P* < .05) was determined using a two-tailed *t* test comparing TGA2.1-NahG with NahG (*p_1_*) and TGA2.1-NahG with NT (*p_2_*). C, Phenotypic differences in PVY-infected leaves of DEX-treated TGA2.1-NahG line 12, NahG, and NT plants at 10 dpi.

SA signaling has proven vital for the establishment of an efficient defense response against potato virus Y (PVY) infection in resistant potato cultivars (Baebler et al., 2014; Lukan et al., 2020). We thus investigated the role of StTGA2.1 in plant immunity using the potato-PVY pathosystem. We generated SA-deficient NahG transgenic potato plants inducibly overexpressing *StTGA2.1* (TGA2.1-NahG) using the glucocorticoid-system (Aoyama and Chua, 1997), in which target gene expression is controlled by external application of dexamethasone (DEX). Three transgenic TGA2.1-NahG lines, showing more than six-fold induction in *StTGA2.1* expression after DEX treatment (Supplemental Figure S2, A–C), were selected for further analysis. We observed that viral replication was significantly reduced in TGA2.1-NahG compared to NahG at 10 days post infection (dpi) (Figure 2, Supplemental Figure S2, D–F). As expected, little to no PVY was detected in NT plants exhibiting a typical resistant phenotype (Baebler et al., 2014). This shows that overexpression of *StTGA2.1* can compensate for the lack of SA in potato immune response to PVY.

### StTGA2.1 retains its dimerization ability and shows a distinct localization pattern

**Figure 3:**
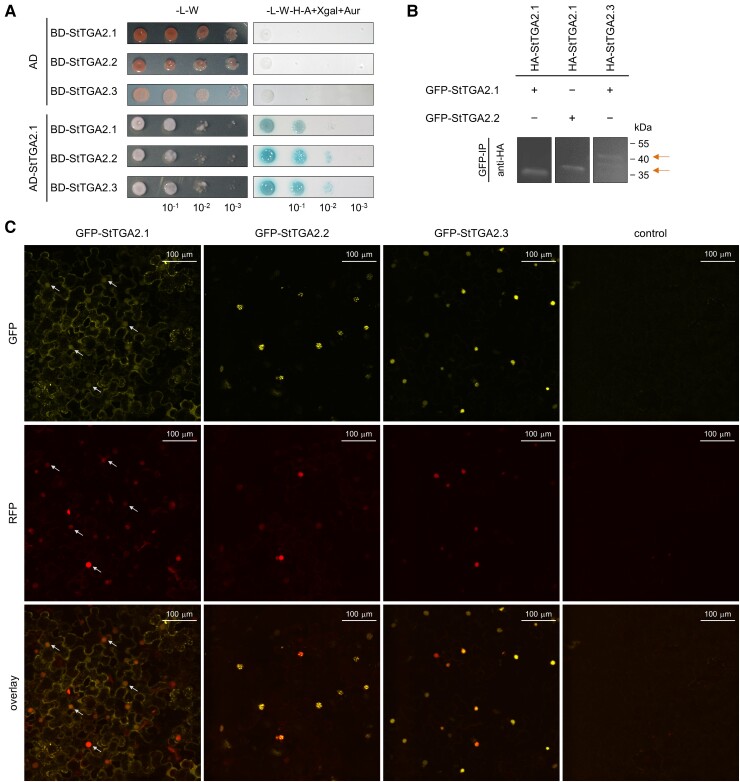
StTGA2.1 can form homodimers, heterodimers, and localizes to diverse cellular compartments. A, StTGA2.1 interactions with itself, StTGA2.2, or StTGA2.3 in the yeast two-hybrid assay. Yeast were cotransformed with bait (BD) and prey (AD) construct combinations and selected on control media without Leu and Trp (-L-W). Positive interactions were determined by yeast growth on selection media without Leu, Trp, His and adenine, with added X-α-galactosidase and Aureobasidin A (-L-W-H-A + Xgal + Aur). B, StTGA2.1 interactions with itself, StTGA2.2, or StTGA2.3 in the coimmunoprecipitation assay. The combination of GFP and HA-tagged proteins expressed in *N. benthamiana* is indicated for each sample (+/−). Positive interactions were determined by detection of immunoprecipitated (GFP-IP) complexes with anti-HA antibodies. Arrows indicate expected bands. In case of HA-tagged StTGA2.3, two bands were detected, likely due to partial protein degradation. Controls are shown in Supplementary Figure S3. C, Subcellular localization of GFP-tagged StTGA2.1, StTGA2.2, and StTGA2.3 (yellow) with Histone 2B-monomeric red fluorescent protein 1 (H2B-RFP) nuclear marker (red) in *N. benthamiana* leaves. The *p19* silencing suppressor was expressed as control. Protein fluorescence is represented as the z-stack maximum projection. Arrows indicate examples of StTGA2.1 nuclear localization. Scale bars, 100 μm.

Protein interaction studies using the yeast two-hybrid assay showed that StTGA2.1 can form both homodimers and heterodimers with StTGA2.2 and StTGA2.3 (*Sotub01g009430*) (Figure 3A), further confirmed by *in planta* coimmunoprecipitation assay (Figure 3B, Supplemental Figure S3, A–C). Additionally, the size-exclusion chromatography (SEC) elution volume of a recombinant His_6_-tagged StTGA2.1 corresponded to the size of a dimer (Supplemental Figure S3, D and E), while chemical cross-linking of a nontagged protein yielded monomers, dimers, and higher order complexes (Supplemental Figure S3F). Overall, these results demonstrate that StTGA2.1 retains protein–protein interaction ability. In addition, we examined whether StTGA2.1 can interact with two potato NPR cofactors, a putative ortholog of AtNPR1, StNPR1 (*Sotub07g011600*), and a putative ortholog of AtNPR3 and AtNPR4, StNPR3/4 (*Sotub02g015550*). Our results showed that StTGA2.1 as well as StTGA2.2 and StTGA2.3 interact with both StNPRs in yeast and that the addition of SA to the media promotes these interactions (Supplemental Figure S4). Thus, the ability to interact with NPR proteins is not perturbed in mini-TGA StTGA2.1.

Subcellular localization of green fluorescent protein (GFP)-tagged StTGA2.1 in the *N. benthamiana* leaf epidermis and mesophyll showed that it can localize to cell nuclei (Figure 3C). StTGA2.1 also showed a distinct localization pattern with intense fluorescence in the cytoplasm, which was enhanced around the chloroplasts (Supplemental Figure S5A). We also detected its fluorescence in the ER and in granular formations of about 0.5–1.0 *μ*m in size (Supplemental Figure S5). In contrast, StTGA2.2 and StTGA2.3 showed predominantly nuclear localization and were organized into subnuclear formations of different sizes within the nuclei (Figure 3C, Supplemental Figure S6).

### Identification of potential StTGA2.1 targets with spatial transcriptomic profiling

To gain insight into the mini-TGA mechanism of action in plant immunity, we examined the expression profile of NahG plants overexpressing *StTGA2.1*. By sampling tissue sections containing lesions and their immediate surrounding area after PVY infection (Supplemental Figure S7A), we were able to follow transcriptomic changes in PVY-responding cells (Lukan et al., 2020). RNA sequencing results showed a regulation of 217 genes due to *StTGA2.1* overexpression in the NahG background (TGA2.1-NahG vs. NahG plants comparison, Supplemental Table S3). However, over 1,800 genes were differentially expressed exclusively in TGA2.1-NahG, when plants were exposed to pathogen infection (Supplemental Figure S7B). Technical validation of the RNA sequencing data by reverse transcription quantitative PCR (RT-qPCR) is shown in Supplemental Table S4.

Gene set enrichment analysis (Supplemental Table S5) enabled us to extract key differences in gene expression at the level of processes or functionally related gene groups (BINs), as they are defined by the MapMan ontology (Ramšak et al., 2014). Important immunity-related or regulatory BINs, enriched in up- or down-regulated genes of PVY- vs. mock-inoculated plants for all three genotypes, are listed in Table 1. Up-regulated genes enriched uniquely in TGA2.1-NahG included isoprenoid metabolism-related genes, PHD finger and PHOR1 transcription factors, and peroxidases (Table 1). On the other hand, the C2C2(ZN) DOF transcription factors were enriched in down-regulated genes in TGA2.1-NahG plants. PVY-regulation of cytokinin and jasmonate metabolism was lost in TGA2.1-NahG compared with the other two genotypes, as was the up-regulated expression of WRKY transcription factors (Table 1).

**Table 1:** Selected functional groups (BINs) enriched in up- or down-regulated genes in TGA2.1-NahG, NahG, and NT plants after PVY infection. FDR corrected *q* value <.05. (+), enriched in up-regulated genes; (−), enriched in down-regulated genes

BIN	Functional group	Size	TGA2.1-NahG	NahG	NT
	Secondary metabolism				
16.1.2	ȃMevalonate pathway	47	+		
16.1.5	ȃTerpenoids	155	+		
16.10	ȃSimple phenols	38			+
	Hormone metabolism				
17.1.3	ȃAbscisic acid-regulated	32	**−**	**−**	
17.2	ȃAuxin	351	**−**	**−**	
17.2.3	ȃAuxin-regulated	287	**−**	**−**	
17.4.1	ȃCytokinin	85		**−**	**−**
17.7	ȃJasmonate	64		+	+
17.8	ȃSalicylic acid	22			+
	Stress—biotic				
20.1.7	ȃPR proteins	208	+	+	+
20.1.7.1	ȃPR-1	33	+		
20.1.7.3	ȃPR-3/PR-4/PR-8/PR-11	44	+	+	+
	Miscellaneous				
26.12	ȃPeroxidases	139	+		
26.21	ȃProtease inhibitor/seed storage/lipid transfer proteins	117		**−**	
	Transcription regulation				
27.3.32	ȃWRKY transcription factors	97		+	+
27.3.63	ȃPHD finger transcription factors	49	+		
27.3.64	ȃPHOR1 transcription factors	21	+		
27.3.8	ȃC2C2(Zn) DOF transcription factors	41	**−**		
	DNA synthesis—chromatin structure				
28.1.3	ȃHistone	83	+	+	+
28.1.3.2	ȃHistone core	76	+	+	+
28.1.3.2.1	ȃHistone core H2A proteins	26	+	+	
28.1.3.2.3	ȃHistone core H3 proteins	20	+	+	+
	Protein degradation				
29.5.11.20	ȃUbiquitin-proteasome	73	+	+	
	Signaling—receptor kinases				
30.2.8.1	ȃLeucine-rich repeat VIII (type 1)	20			**−**
30.2.16	ȃ*Catharanthus roseus*-like RLK1	79			+
30.2.17	ȃDUF26	94			+
30.2.19	ȃLegume-lectin	38	+		+
30.2.99	ȃMiscellaneous	206	+		+

### StTGA2.1 and StTGA2.3 activate the class III peroxidase StPRX07

**Figure 4:**
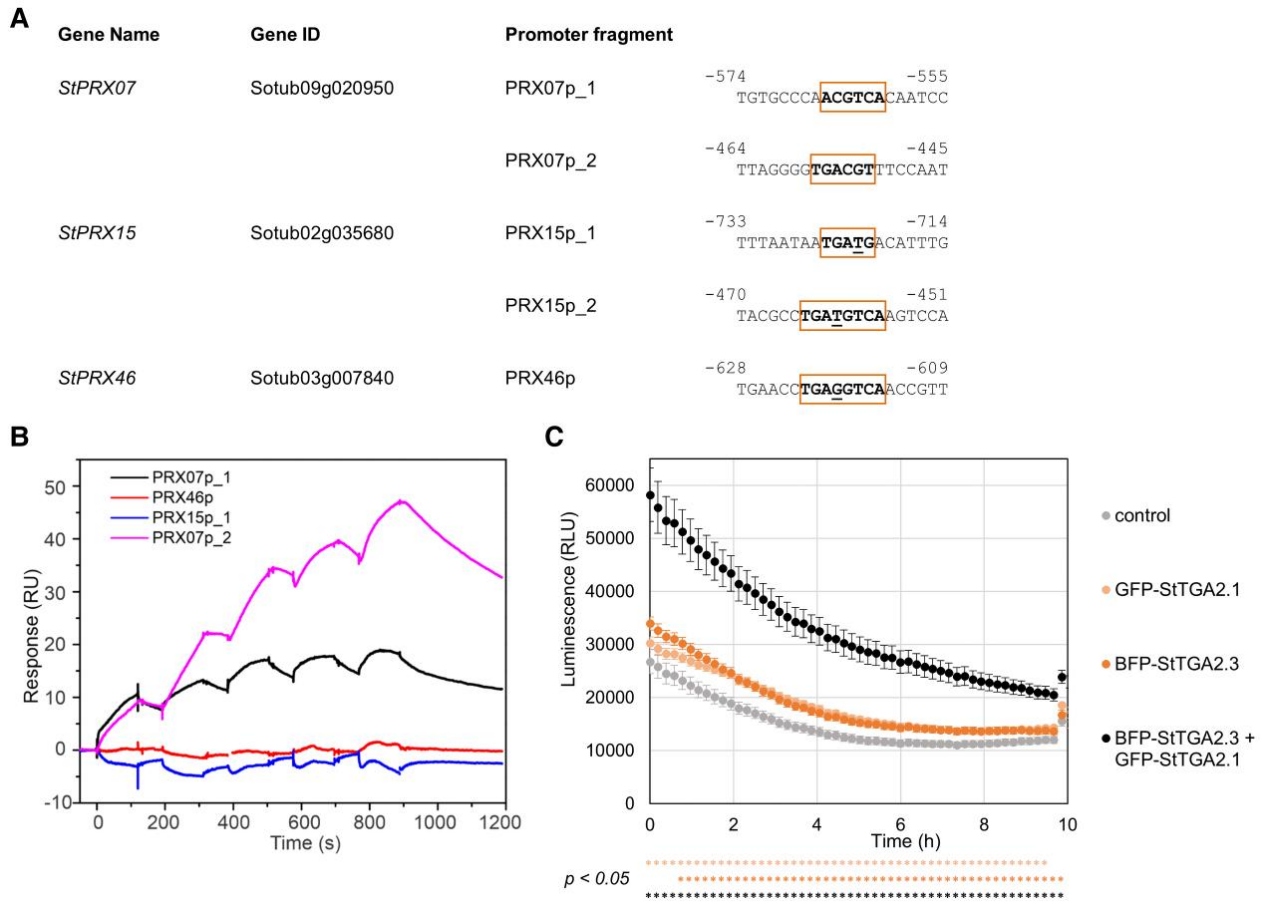
StTGA2.1, together with its interactor StTGA2.3, activates expression of StPRX07. A, TGA-binding motifs in selected *StPRX* promoters. The predicted motifs are boxed and the nucleotides, differing from the core TGACG(T) sequence, its reverse complement or the TGACGTCA palindrome, are underlined. Numbers indicate position upstream of transcription start site. B, Surface plasmon resonance results, showing the interaction between StTGA2.3 and chip-immobilized PRX15p_1, PRX46p, PRX07p_1, or PRX07p_2 DNA fragments, bound to the chip at ∼38, 41, 65, or 53 response units (RU), respectively. Representative sensorgrams are shown. C, Transactivation assay results, showing *in planta StPRX07* promoter activation by GFP-tagged StTGA2.1 (light orange), BFP-tagged StTGA2.3 (dark orange) or a combination of both (black). BFP or GFP-tagged controls and their combination (control) were used to detect the basal promoter activity (grey). Average values ± SE of 18 biological replicates in the first 10 h of measurement are shown. Significance (*P* < .05) was determined using a two-tailed *t* test and is shown below the response curve for GFP-tagged StTGA2.1 (light orange), BFP-tagged StTGA2.3 (dark orange), or a combination of both (black) compared with control. The experiment was repeated twice with similar results (Supplemental Figure S8C). RLU, relative light units.

As the expression of several peroxidases was up-regulated after *StTGA2.1* overexpression in PVY-infected NahG plants (Table 1, Supplemental Table S5), we recognized them as potential direct targets of StTGA2.1. To test our hypothesis, we selected three class III peroxidases (StPRX, Supplemental Table S6), *StPRX07* (*Sotub09g020950*), *StPRX15* (*Sotub02g035680*), and *StPRX46* (*Sotub03g007840*), which were up-regulated in TGA2.1-NahG compared with NahG (Supplemental Tables S3 and S7), for further analysis. Analysis of their promoter regions revealed predicted TGA-binding motifs between 450 and 750 bp upstream of the transcription start site (Figure 4A).

To investigate the ability of StTGAs to bind these motifs, we first tested whether the StTGA2.3 and StTGA2.1 proteins could bind to four candidate DNA fragments from *StPRX* promoter regions, PRX07p_1, PRX07p_2, PRX15p_1, and PRX46p (Figure 4A), using surface plasmon resonance. Titration of a recombinant StTGA2.3 over chip-immobilized PRX07p_1 and PRX07p_2 fragments, carrying the predicted TGA-binding motifs of the *StPRX07* promoter, resulted in a dose-dependent increase in response, compared to reference (Figure 4B). Interaction with PRX15p_1 and PRX46p fragments was negligible (Figure 4B). As predicted by the absence of the basic region, we did not measure any interaction between the His_6_-tagged StTGA2.1 and the tested DNA (Supplemental Figure S8A). These results suggest that StTGA2.3, but not StTGA2.1, binds specifically to the TGA-binding motifs in the *StPRX07* promoter. Furthermore, titration of StTGA2.3 premixed with StTGA2.1 over PRX07p_1 and PRX07p_2 fragments resulted in higher responses compared with StTGA2.3 alone, whereas this was not the case for PRX15p_1 (Supplemental Figure S8B). These results support the formation of a StTGA2.1-StTGA2.3 complex at the *StPRX07* regulatory region.

Finally, we tested the ability of StTGA2.3 and StTGA2.1 to activate the *StPRX07* promoter *in planta*, using a transient transactivation assay (Lasierra and Prat, 2018). For this purpose, the 2.95 kb long promoter region upstream of the *StPRX07* start codon, containing both predicted TGA-binding motifs, was fused to a luciferase (*LucF*) coding sequence. GFP-tagged StTGA2.1 and BFP-tagged StTGA2.3 were then coexpressed with the reporter construct, confirmed by confocal microscopy. Coexpression of the reporter construct with StTGA2.3 induced the expression of *StPRX07*::*LucF* by approximately 20% compared with basal promoter activity, whereas coexpression with StTGA2.1 resulted in only minor induction (Figure 4C, Supplemental Figure S8C). In contrast, more than two-fold induction in promoter activity was observed when coexpressed with both StTGA2.1 and StTGA2.3, compared to control, meaning the induction was about three to four-times stronger when both StTGAs were overexpressed (Figure 4C, Supplemental Figure S8C). These results indicate that strong activation of *StPRX07* promoter is achieved only when both StTGA2.3 and StTGA2.1 are present.

### StTGA2.1 N terminus likely contributes to protein interactions and alters TGA binding to DNA

**Figure 5:**
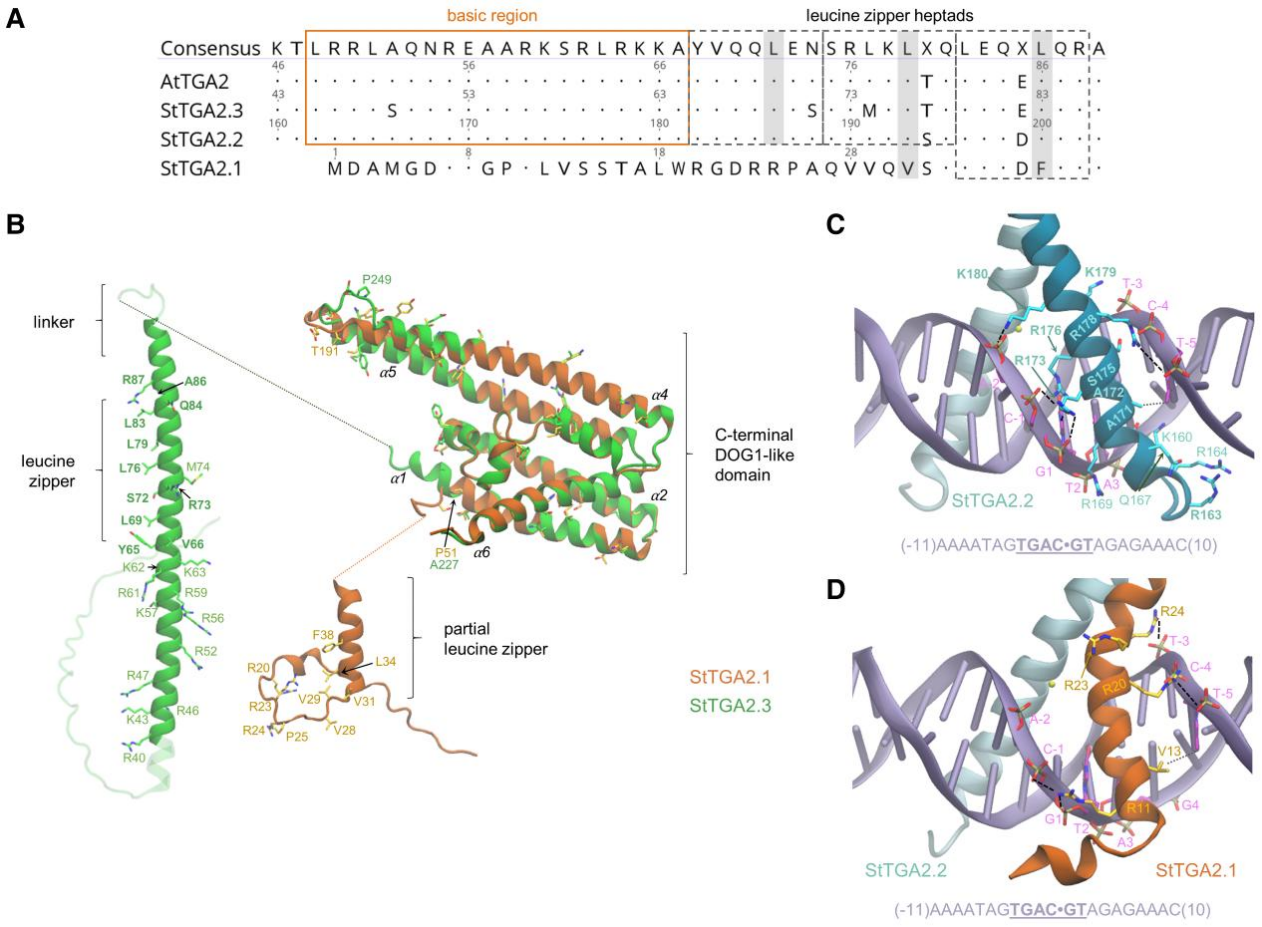
Comparative structural analysis and simulations of StTGA2.1 N terminus interactions with StTGA2.2 and StTGA2.3 bZIP domains. A, Protein sequence alignment of StTGA2.1 N terminus with AtTGA2, StTGA2.2, and StTGA2.3 bZIP domains. The basic region (orange box) and the leucine zipper heptads (grey dashed boxes) are indicated. Conserved amino acids, in respect to the consensus sequence, are marked with dots. StTGA2.1 contains hydrophobic residues (Val31 and Phe38) in two out of three Leu positions in the heptads (grey) and has a completely conserved third heptad. B, Molecular architectures of StTGA2.1 (orange) and StTGA2.3 (green). The StTGA2.3 bZIP domain (aa 40–95), and StTGA2.1 N terminus (aa 1–45) are shown. The C-terminal region is highly conserved between StTGA2.1 (aa 47–240), StTGA2.2 (aa 222–446), and StTGA2.3 (aa 105–327). Helices in the C-terminal part are labelled (α1–α6). Amino acid residues that are discussed in this study are represented as liquorice and labelled for StTGA2.1 (orange) and StTGA2.3 (green). Those forming persistent contacts in the leucine zipper, according to MD simulations, are shown in bold (green). Basic amino acid residues that may contribute to DNA-binding, in the regions aa 40–63 of StTGA2.3 and aa 20–28 of StTGA2.1, are depicted and labelled. Fully nonconservative substitution sites in the putative DOG1 domains are also represented as liquorice. C, Representative snapshot of the MD simulations of the DNA-bound StTGA2.2 homodimer and D, StTGA2.2-StTGA2.1 heterodimer. The DNA double helix is represented in violet. The DNA sequence is shown at the bottom and the binding site core is underlined. A dot is used as a reference at the center of the sequence for numbering the nucleotide residues. StTGA2.1 (orange) and StTGA2.2 (cyan) are represented as cartoon. Salt bridges and hydrogen bonds between protein and DNA are indicated with black dashed lines. Hydrophobic contacts are indicated as dotted lines. Amino acid residues forming persistent interactions are labelled in bold. The presence of a hexacoordinated Mg^2+^ was assumed (yellow sphere), based on its importance for CREB-bZIP (Moll et al., 2002).

Comparative structural analysis using AlphaFold (AF) (Jumper et al., 2021) revealed important singularities in the molecular architecture of StTGA2.1, mostly contained in its N terminus. In the AF models of StTGA2.2 and StTGA2.3, the intrinsically disordered N terminus is followed by an α-helical bZIP domain, which includes several basic residues and three heptads that comprise the leucine zipper (Figure 5, A and B, Supplemental Figure S9). In contrast, StTGA2.1 has a short N terminus with low helical propensity due to the Pro25 α-helix breaker, harboring only the basic residues Arg20, Arg30, and Arg24, and lacking the first and most of the second leucine zipper heptad (Figure 5, A and B). The conserved hydrophobic residues Leu34, Val31, and Phe38 may contribute to protein dimerization by forming a partial zipper that could be stabilized by the hydrophobic Val28 and Val29. Persistent contacts identified in MD simulations of StTGA2.2 and StTGA2.3 homo- and heterodimers are preserved in StTGA2.1 and involve the said hydrophobic residues (Figure 5B, Supplemental Figure S10).

We then inquired if the StTGA2.1 N terminus binds to DNA. Based on prior knowledge of AtTGA2 and its cognate TGACG motif (Boyle et al., 2009), we modeled the DNA-bound StTGA2.2-StTGA2.2 and StTGA2.2-StTGA2.1 dimers and, via MD simulations, identified the key DNA-binding residues (Figure 5, C and D, Supplemental Tables S8 and S9). Persistent interactions in the StTGA2.2 homodimer mostly correspond to salt bridges, formed between the bZIP basic residues and the DNA phosphate groups, and do not explain the StTGA2.2 motif-binding specificity. In contrast, sequence specificity is provided by hydrophobic contacts between the StTGA2.2 Ala172 residue and the DNA T2 methyl group or between Ala171 and T-4 or T-5. Indeed, most of the predicted TGA-binding motifs (Figure 4A) have a thymine or adenine in these positions. While the contacts involving StTGA2.2 in the StTGA2.1-StTGA2.2 heterodimer are highly preserved, the StTGA2.1–DNA interactions are dramatically reduced, with only Arg11, Arg20, and Arg24 forming persistent contacts with the DNA phosphate groups (Figure 5D, Supplemental Table S9). Moreover, the StTGA2.1 partial zipper positions the last basic residue (Arg24) more distant to the DNA compared to StTGA2.2 (Lys180), breaking the protein–DNA complex symmetry.

Another important singularity of StTGA2.1 is that its partial zipper connects directly to the putative DOG1 domain, while the two domains are connected by a 13 aa peptide linker in StTGA2.2 and StTGA2.3. This may greatly influence its interdomain conformational flexibility. Pro51 in StTGA2.1 (Ala227 and Ala110, in StTGA2.2 and StTGA2.3, respectively), disrupts the DOG1 α-helix 1 (α1) and forms a shorter, disordered linker that could somehow compensate for this absence. These results indicate that the compact molecular architecture of StTGA2.1, which causes an asymmetric distribution of basic residues in the StTGA2.1–StTGA2.2 heterodimer, substantially distorts the DNA conformation near the binding site, supporting strong promoter activation upon binding of the heterodimer compared with binding of the homodimer.

## Discussion

TGAs are involved in modulation of various cellular processes, acting as positive or negative regulators of gene expression (Gatz, 2013). Their structural features provide the basis for their functional variability, defining their subcellular localization, target recognition, DNA-binding, as well as their ability to form dimers, oligomers, or interact with other proteins (Tomaž et al., 2022). In Arabidopsis, all 10 AtTGAs share a highly conserved bZIP domain, essential for establishing specific interactions with DNA. Here, we report on the structural-functional relationship of a mini-TGA from potato, StTGA2.1, which lacks a full bZIP but still acts at target gene activation. Mini-TGAs were identified in potato (this study), tomato (Hou et al., 2019; Lemaire-Chamley et al., 2022), and strawberry (Feng et al., 2020), but not in Arabidopsis.

Homo- and heterodimerization of StTGA2.1 (Figure 3, A and B, Supplemental Figure S3), which contains only a part of an already short and presumably unstable TGA zipper region (Deppmann et al., 2004), corroborates the findings of Boyle et al. (2009), who established that the leucine zipper is not crucial for dimerization of AtTGA2 (Boyle et al., 2009). Instead, StTGA2.1 dimerization is likely mediated by interactions involving its C-terminal region, previously reported to contain a dimer stabilization region (Katagiri et al., 1992). In line with this, the recent cryo-EM structure of an NPR1-AtTGA3 complex revealed the formation of stable homodimer contacts between two AtTGA3 C-terminal regions (Kumar et al., 2022). StTGA2.1 nuclear localization allows its role in gene regulation; however, its unusually broad localization pattern (Figure 3C, Supplemental Figure S5) suggests that it might perform different tasks, as has been shown for other plant transcription factors with intrinsically disordered regions (Powers et al., 2019). TGAs also interact with different proteins present in both nuclei and cytoplasm, including NPR cofactors (Boyle et al., 2009; Ding et al., 2018), which was already confirmed for StTGA2.1 (Supplemental Figure S4), glutaredoxins (Li et al., 2009), and calmodulins (Popescu et al., 2007). Thus, StTGA2.1 could modulate the function of these partners by sequestering them into inactive complexes, as was proposed for AtTGA2 and NPR1 (Fan and Dong, 2002).

Multiple functions of StTGA2.1 are supported also by the diversity of detected transcriptional changes following exposure to pathogen infection (Table 1, Supplemental Tables S3 and S5). StTGA2.1 may directly affect the activity of several transcriptional regulators and its effects on gene expression are likely amplified by further secondary regulation. Thus, we hypothesize StTGA2.1 is an important player in shaping the transcriptional landscape during infection. Interestingly, our results show StTGA2.1 improves potato immunity even in the absence of SA, even though clade II TGAs are known for their regulatory role in the SA pathway (Zhang et al., 2003; Zhou and Zhang, 2020). Our results thus indicate that the TGA mechanism of action in potato may differ substantially from that in Arabidopsis. It is important to note that *StTGA2.1* overexpression in SA-deficient plants may not reflect its function in nontransgenic potato completely, as several parts of immune signaling are under the control of SA and are thus not fully functional in our transgenic plants. We have, however, focused on overexpression in a SA-deficient background to check whether resistance towards PVY can be improved. Overexpression of *StTGA2.1* in a nontransgenic background would not result in observable phenotypic differences, because the plants are already fully resistant to PVY. Furthermore, as we did not find *StTGA2.1* transcriptionally regulated during PVY infection in nontransgenic plants (Supplemental Tables S2, S4, and S7), the preparation of knockout nontransgenic plants is unlikely to affect plant immunity.

Several studies have evaluated the influence of clade II TGA dominant-negative bZIP mutants on plant immunity during bacterial infection, leading to contradictory results (Niggeweg et al., 2000; Pontier et al., 2001; Fan and Dong, 2002). Reactive oxygen species (ROS) act as signaling molecules in biotic stress (Bleau and Spoel, 2021). Early ROS production is central to plant defense and TGAs have previously been associated with cellular redox control, physically interacting with or regulating the expression of CC-type glutaredoxins (Ndamukong et al., 2007; Li et al., 2009; Hou et al., 2019). Furthermore, clade II TGAs modulate the expression of glutathione-S-transferases in ROS-processing responses to UV-B stress (Herrera-Vásquez et al., 2021), while clade IV AtTGAs are regulated by flg22-induced ROS production (Noshi et al., 2016). Here we show that the synergistic activity of TGAs regulates the expression of yet another group of enzymes involved in ROS-metabolism, the class III peroxidases (Figure 4, B and C, Supplemental Figure S8). Class III peroxidases are heme-containing glycoproteins, secreted to the apoplast or localized in vacuoles (Almagro et al., 2009). Among them, AtPRX33 and AtPRX34 proved vital for apoplastic ROS production in response to flg22 and elf26 (Daudi et al., 2012). Most of the StPRX protein sequences from the peroxidase functional group (Ramšak et al., 2014) contain predicted secretory signal peptides (Supplemental Table S6), indicating StTGA2.1 could affect apoplastic ROS production in plant defense.

Transcription factor cooperativity is essential in eukaryotic transcription regulation and can arise through various mechanisms, involving protein–protein and/or protein–DNA interactions (Morgunova and Taipale, 2017). For example, the interaction between two NPR1 proteins bridges two AtTGA3 homodimers bound to separate DNA-binding motifs, creating a complex with an AtTGA3_2_-NPR1_2_-AtTGA3_2_ stoichiometry (Kumar et al., 2022). Previous studies have shown that TGA mutants, impaired in DNA-binding through diverse modifications of the bZIP domain, prevent DNA-binding of wild type homologs (Rieping, 1994; Niggeweg et al., 2000; Pontier et al., 2001), which somewhat opposes the cooperative activation via an StTGA2.1-StTGA2.3 complex. Compared to homodimers of its putative paralogs, our MD simulations suggest that the asymmetrical distribution of basic residues in the bZIP-like domain in the StTGA2.1-StTGA2.2 heterodimer substantially distorts the DNA conformation near its binding site (Figure 5). We hypothesize that StTGA2.1 dramatically affects the overall conformation of the regulatory complex due to its compact molecular architecture.

In conclusion, we show that, although mini-TGAs are not able to bind DNA on their own, their unusual structure supports diverse functionalities, such as allowing the induction of class III peroxidases in immune signaling. We thus provide evidence that truncation in evolution of genes does not necessary lead to a loss-of-function phenotype. Instead, additional functions can be attained. Through this, we shed additional perspective on immune signaling in nonmodel species, as Arabidopsis does not encode such proteins.

## Materials and methods

### *In silico* sequence and structural analysis

TGA transcription factor orthologs from potato (*Solanum tuberosum*) were identified based on ortholog information included in the GoMapMan database (Ramšak et al., 2014). The initial list was further pruned based on protein sequence alignments created with Geneious Alignment in Geneious Prime 2020.1.1 (https://www.geneious.com/) and BLAST results to exclude technical errors of ortholog detection and sequencing. Identified StTGAs are listed in Supplemental Table S1. Basic protein information was calculated using the ExPASy ProtParam tool (Gasteiger et al., 2005). Protein domain prediction was performed with ExPASy Prosite (de Castro et al., 2006). Protein sequences of SlTGAs (Hou et al., 2019; Lemaire-Chamley et al., 2022) were retrieved from the Sol Genomics Network (Fernandez-Pozo et al., 2015) and sequences of AtTGAs from The Arabidopsis Information Resource (Berardini et al., 2015).

For the phylogenetic analysis, the sequences were aligned with MAFFT (Katoh and Standley, 2013), using the L-INS-I iterative refinement method, and the alignment used for a maximum-likelihood phylogenetic tree construction in MEGA-X (Kumar et al., 2018), using the Jones-Taylor-Thorton matrix-based model (Jones et al., 1992) and 1,000 bootstrap repetitions. The rice (*Oryza sativa*) OsTGA2.1 (Chern et al., 2001) protein sequence (Q7X993) was retrieved from UniProtKB (https://www.uniprot.org/) and used as tree root. For sequence similarity visualization, the protein sequences were aligned with Geneious Alignment in Geneious Prime 2020.1.1 (https://www.geneious.com/). Potato peroxidases were identified with protein sequence BLAST against the RedoxiBase database (Savelli et al., 2019) and the secretory signal peptides were predicted with SignalP 5.0 (Almagro Armenteros et al., 2019) (Supplemental Table S6). Predictions of transcription factor binding motifs in promoter sequences were performed with TRANSFAC (Matys et al., 2006) and predictions of transcription start sites with TSSFinder (de Medeiros Oliveira et al., 2021).

Structural models of StTGA2.1, StTGA2.2, and StTGA2.3 were generated with AlphaFold (Jumper et al., 2021). The top-ranked models were selected. The VMD (Visual Molecular Dynamics, version 1.9.4a48) molecular visualization program was used for visual analysis and structural alignment of protein models.

### MD simulations

The initial homo- and heterodimeric configurations of StTGA2.1-StTGA2.2, StTGA2.2-StTGA2.2, and StTGA2.2-StTGA2.3 N-terminal fragments were defined using the crystal structure of CREB-bZIP-CRE (PDB id: 1DH3) as template (Schumacher et al., 2000). Corresponding amino acid changes to the template were done using the VMD psfgen plugin, preserving the coordinates of the backbone and C_β_ atoms. StTGA2.1, StTGA2.2, and StTGA2.3 are truncated, keeping the amino acids 1–43, 159–206, and 42–89, respectively. The N termini of StTGA2.2 and StTGA2.3 are capped with N-methylamide and the C termini of the three proteins with acetyl. For simulations of DNA-bound StTGA2.1-StTGA2.2 and StTGA2.2-StTGA2.2, the DNA fragment from the template crystal structure was kept and the nucleotides were modified using psfgen. The final DNA sequence corresponds to the TGACGT motif, complementary to the linker scan 5 element and its adjacent regions of Arabidopsis (*Arabidopsis thaliana*) *PR-1* promoter *as-1*-like sequence (Lebel et al., 1998) (Figure 5, C and D). The crystal Mg^2+^ cation and the six coordinated water molecules were kept.

GROMACS-2020 (Abraham et al., 2015) was used to prepare inputs and run MD simulations. The simulation boxes were generated as an octahedron, defining a solvation layer of 10 Å minimum thickness around the molecular complex. NaCl of 0.15 M was used to establish electroneutrality. Protonation states were defined for pH 7.0. Amber ff99SB (Lindorff-Larsen et al., 2010) and ff14SB9 (Maier et al., 2015) were used to describe the protein in the free and DNA-bound TGA dimers, respectively, and PARMBSC1 (Ivani et al., 2015) was used to describe the DNA. TIP4P-D (Piana et al., 2015) or simple point charge (SPC) (Berendsen et al., 1981) was used to describe water molecules in the simulations of the free and DNA-bound TGA dimers, respectively. CHARMM-formatted topology and parameter files were converted to GROMACS input files using the VMD plugin TopoGromacs (Vermaas et al., 2016).

The MD simulations were performed on the Summit supercomputer at the Oak Ridge Leadership Computing Facility. Energy minimization was performed for all systems with steepest descent. Periodic electrostatic interactions were treated with the particle mesh Ewald method (Darden et al., 1993). LINCS (Hess et al., 1997) was used to constrain bonds involving hydrogen atoms.

Similar protocols of simulation were applied for the free and DNA-bound TGA dimers. Preceding the classical simulations, we performed long equilibration runs of 315 ns as part of our protocol adapted from the MD simulation-based method of structural refinement described by Heo et al. (2021). In this protocol, potential sampling is accelerated with hydrogen mass repartitioning and by applying fairly high temperatures. Weak position-restraint potentials were applied for minimum bias and to compensate for the high thermal energy. Velocity Langevin dynamics was performed using a friction constant of 1 ps^−1^. During the equilibration phase, position restraints applied to C_α_ atoms in the leucine heptads were gradually released and the temperature gradually increased, reaching the maximum of 360 K (Supplemental Table S10). After long sampling at 340 and 320 K, a final phase of equilibration is conducted at 298.15 K, the temperature of the following production runs. During the final equilibration phase, flat-bottom harmonic-restraint potentials were applied, using a force constant of 0.25 kcal/mol/Å^2^ and a flat-bottom width of 4 Å. To adjust box size, part of the equilibration phase was conducted in the *NpT* ensemble, using the Berendsen barostat (Berendsen et al., 1984) applying a compressibility of 4.5 × 10^−5^ bar^−1^ and a time constant of 1.0 ps. In the final phase of equilibration, the atomic velocities were assigned from a Maxwell–Boltzmann distribution using random numbers of seed. The production runs of free and DNA-bound dimers consisted of five unbiased independent simulations of 128 and 200 ns, respectively. The position-restraint potential applied to Mg^2+^ and its coordinated water molecules was kept during these simulations.

For simulation analysis, the VMD plugin Hbonds was used to count hydrogen bonds formed during the production runs. The geometric criteria adopted are a cut-off of 3.0 Å for donor–acceptor distance and 20° for acceptor–donor-H angle. In Figure 5, C and D, salt bridges and hydrogen bonds between protein–DNA occurring during more than 10% of the simulation time are shown. Persistent contacts were identified using the VMD plugin Timeline. In Figure 5, C and D, amino acid residues involved interactions or hydrophobic contacts persisting for more than 30% of the simulation time are shown. Grace was used for plots (https://plasma-gate.weizmann.ac.il/Grace/).

### Plant material

Potato nontransgenic cultivar Rywal (NT) and Rywal-NahG (NahG), a transgenic line impaired in SA accumulation due to salicylate hydroxylase expression (Baebler et al., 2014), were used in this study. Plants were propagated from stem node tissue cultures and transferred to soil 2 weeks after node segmentation, where they were kept in growth chambers under controlled environmental conditions at 22/20°C with a long-day (16 h) photoperiod of light (light intensity 4,000 lm/m^2^) and 60%–70% relative humidity. *Nicotiana benthamiana* plants were grown from seeds and kept in growth chambers under the same conditions.

### DNA constructs

Full-length coding sequences (cds) of *StTGA2.1*, *StTGA2.2*, *StTGA2.3*, *StNPR1*, and *StNPR3/4* were amplified from potato cultivar Rywal cDNA and inserted into the pJET1.2/blunt cloning vector using the CloneJET PCR Cloning Kit (Thermo Scientific, USA), following the manufacturer's instructions.

The selected genes were subsequently cloned into pENTR D-TOPO vector using pENTR Directional TOPO Cloning Kit (Invitrogen, USA) and recombined through LR reaction using the Gateway LR Clonase II Enzyme Mix (Invitrogen, USA) into several Gateway destination vectors (VIB, Belgium). For coimmunoprecipitation experiments, localization studies, and transactivation assays, the *StTGA2.1*, *StTGA2.2*, and *StTGA2.3* cds were inserted into pH7FWG2 and pJCV52 expression vectors (Karimi et al., 2002) to produce proteins with C-terminal enhanced GFP and hemagglutinin A (HA) fusions, respectively. For transactivation assays, *StTGA2.3* cds was fused with the *mTagBFP2* cds (from Addgene plasmid # 102638) (Stark et al., 2018), to produce a protein with a C-terminal blue fluorescent protein (BFP) tag, prior to cloning into pENTR D-TOPO vector (Invitrogen, USA) and subsequently recombined into the pK7WG2 vector (Karimi et al., 2002). A short linker encoding six Gly residues was introduced between the *StTGA2.3* and *BFP* sequence. *BFP* fused with a short sequence encoding an N7 nuclear localization signal (Ghareeb et al., 2016) (N7-BFP) was recombined into pK7WG2 (Karimi et al., 2002) as control.

For overexpression experiments, the *StTGA2.1* cds was amplified with primers harboring *XhoI* and *Spe*I restriction enzyme cleavage sites and inserted into the pTA7002 vector (Aoyama and Chua, 1997), enabling glucocorticoid-inducible gene expression *in planta*, through restriction–ligation cloning.

For the yeast two-hybrid assays, the cds of *StTGA2.1*, *StTGA2.2*, *StTGA2.3*, *StNPR1*, and *StNPR3/4* were amplified and inserted into the pGBKT7 (bait) yeast expression vector through *in vivo* cloning with Matchmaker Gold Yeast Two-Hybrid System (Clontech, USA), to produce proteins with an N-terminal Gal4 DNA-binding domain. *StTGA2.1*, *StNPR1*, and *StNPR3/4* were inserted also into the pGADT7 (prey) vector (Clontech, USA), to produce proteins with an N-terminal Gal4 activation domain, using the same cloning system.

Promoter sequences of *StPRX07*, *StPRX15*, and *StPRX46* were amplified from potato cultivar Rywal genomic DNA and inserted into the pENTR D-TOPO vector (Invitrogen, USA). The *StPRX07* promoter sequence was subsequently recombined through LR reaction into the pGWB435 Gateway vector (Nakagawa et al., 2007), as described above, inserting the promoter upstream of a luciferase reporter (*LucF*).

For recombinant protein production, the *StTGA2.1* cds was inserted into the pMCSG7 bacterial expression vector (Eschenfeldt et al., 2009) by ligation-independent cloning (Aslanidis and Jong, 1990) to produce a protein with an N-terminal hexahistidine (His_6_) tag. The cds of *StTGA2.3* was amplified using primers, enabling the digestion–ligation reaction with the *Bsa*I restriction enzyme. Three silent mutations were introduced into its sequence, to remove two native *Bsa*I restriction sites. The amplified fragment was subsequently ligated into the pEPQD0KN0025 acceptor backbone (Addgene plasmid #162283) (Dudley et al., 2021), together with pEPQD0CM0030 (Addgene plasmid #162312) (Dudley et al., 2021), which adds an additional GS peptide to the protein C terminus.

All primer pairs used in the cloning procedure are listed in Supplemental Table S11. Sequence verification was performed with Sanger sequencing (Eurofins Genomics, Germany).

### Transient expression assays

Homemade electrocompetent *Agrobacterium tumefaciens* GV3101 cells were transformed with prepared constructs by electroporation. Transformants were used for agroinfiltration of the bottom three fully developed leaves of 3–4-weeks-old *N. benthamiana* plants, as described previously (Lazar et al., 2014). In cases of cotransformation with agrobacteria carrying different constructs, the 1:1 ratio was applied. An equal volume of agrobacteria carrying *p19* silencing suppressor (kindly provided by prof. Jacek Hennig, PAS, Poland) was added to the mixture. Agrobacteria carrying *p19* only were used as controls.

### Confocal microscopy

Protein fluorescence was visualized 3–5 d after transient *N. benthamiana* transformation. For protein localization, the Leica TCS SP5 laser scanning confocal microscope mounted on a Leica DMI 6000 CS inverted microscope with an HC PL FLUOTAR 10× objective or HCX PL APO lambda blue 63.0 × 1.40 oil-immersion objective (Leica Microsystems, Germany) was used, using the settings described previously (Lukan et al., 2018b). The Histone 2B-monomeric red fluorescent protein 1 (H2B-RFP) nuclear marker (Federici et al., 2012) was used to visualize cell nuclei. For coimmunoprecipitation and transactivation assays, the protein fluorescence was confirmed with the Leica TCS LSI macroscope with Plan APO 5× and 20× objectives (Leica Microsystems, Germany), using the settings described previously (Lukan et al., 2018a). The green, blue or red fluorescent protein fluorescence was excited using 488 nm, 405 and 543 nm laser lines, respectively. The emission was measured in the window of 505–520 nm for GFP, 450–465 nm for BFP, 570–630 nm for H2B-RFP and 690–750 nm for autofluorescence. In Figure 3C, gain 550–665 was used for measuring RFP emission, gain 500–800 for measuring GFP emission and gain 550 for measuring autofluorescence. In Supplemental Figures S5 and S6, gain 675–920 was used for measuring RFP emission, gain 500–800 for measuring GFP emission and gain 640–740 for measuring autofluorescence. Laser intensity was set to 60% in all figures. The Leica LAS AF Lite software (Leica Microsystems, Germany) was used for image processing.

### Yeast two-hybrid assay

Bait (containing *StTGA2.1*, *StTGA2.2*, *StTGA2.3*, *StNPR1* or *StNPR3/4* cds), and prey (containing *StTGA2.1*, *StNPR1* or *StNPR3/4* cds) construct combinations were transformed into the Y2H Gold strain using the Matchmaker Gold Yeast Two-Hybrid System (Clontech, USA) and the transformants selected on control SD media without Leu and Trp (-L-W). Interactions were analyzed on selection SD media without Leu, Trp, His and adenine, with added X-α-Gal and Aureobasidin A (-L-W-H-A + Xgal + Aur). The proteins were tested for autoactivation through cotransformation of bait constructs with an empty prey vector. To evaluate the strength of interaction, saturated yeast culture dilutions (10^−1^, 10^−2^ and 10^−3^) were spotted onto selection media. To evaluate the effect of SA on the strength of interaction, the dilutions were spotted onto selection media containing 0.1 mM or 1.0 mM SA.

### Coimmunoprecipitation assay

HA or GFP-tagged StTGA2.1, StTGA2.2 and StTGA2.3 were transiently expressed in *N. benthamiana* leaves in different combinations. The empty pB7WGF2 vector (Karimi et al., 2002), expressing the GFP protein, was used as control. The fluorescence of GFP and GFP-tagged proteins was confirmed with confocal microscopy after 4 d. Total proteins were extracted from ∼500 mg leaf material with immunoprecipitation (IP) buffer, containing 10 mM Tris-HCl, pH 7.5, 150 mM NaCl, 2 mM MgCl_2_, 1 mM dithiothreitol and 1x EDTA-free Protease Inhibitor Cocktail (Roche, Switzerland), followed by 1 h incubation with GFP-Trap Magnetic Agarose beads (ChromoTek, Germany) at 4°C. The beads were washed three times with IP buffer and eluted into SDS-PAGE loading buffer, containing 100 mM Tris-HCl, pH 6.8, 4% (w/v) SDS, 0.2% (w/v) bromophenol blue, 20% (v/v) glycerol and 200 mM dithiothreitol. The immunoprecipitated proteins and protein extracts were analyzed by SDS-PAGE and Western blot, using anti-GFP (diluted 1:3,000 or 1: 5,000, Invitrogen, USA) and anti-HA (diluted 1:1,000, ChromoTek, Germany) antibodies.

### Generation of StTGA2.1 overexpression plants

Transgenic TGA2.1-NahG plants were obtained by stable transformation of the Rywal-NahG potato genotype (Baebler et al., 2014). Electrocompetent *A. tumefaciens* strain LBA4404 was electroporated with the pTA7002 vector (Aoyama and Chua, 1997) carrying the *StTGA2.1* cds, as described above. Agrobacteria were used for stable transformation of sterile plantlet stem internodes from tissue culture, as described previously (Lukan et al., 2022). Plantlets grown on regeneration media plates with hygromycin selection were sub-cultured in order to generate independent transgenic lines. Transgenic lines were confirmed with PCR (Supplemental Table S11). Lines 7, 12, and 13 were selected for further analysis.

### Virus inoculation and plant treatments

Three- to four-weeks-old potato plants were inoculated with GFP-tagged infectious PVY clones PVY^N605^-GFP (Rupar et al., 2015) or PVY^N605^(123)-GFP (Lukan et al., 2022) or mock inoculum, as described previously (Baebler et al., 2009). To induce gene overexpression, plants were treated with DEX foliar spray solution containing 30 *μ*M DEX and 0.01% (v/v) Tween-20 or control spray solution without DEX (control), 3 h prior to virus inoculation, 3 h after virus inoculation, and every day post inoculation until sampling.

### Gene expression analysis with RT-qPCR

For gene expression analysis, total RNA isolation and RT-qPCR were performed as described previously (Baebler et al., 2014). DEX-induced *StTGA2.1* overexpression in fully developed leaves of TGA2.1-NahG transgenic lines was confirmed 3 h after DEX treatment using a RT-qPCR assay targeting *StTGA2.1* cds. The leaves of three DEX-treated plants and two or three nontreated plants were sampled, one leaf per plant. For PVY abundance analysis, PVY-infected leaves of DEX-treated TGA2.1-NahG, NahG and NT genotypes were sampled at 3, 5, and 7 dpi or 3, 7, and 10 dpi. For each genotype and treatment, three plants were analyzed, sampling one leaf per plant per dpi. PVY abundance and *StTGA2.1* expression were quantified using two sample dilutions and a relative standard curve method by normalization to the endogenous control *StCOX1* with quantGenius (http://quantgenius.nib.si) (Baebler et al., 2017). A two-tailed *t* test was used to compare treatments, when applicable. The RT-qPCR analysis was performed for TGA2.1-NahG transgenic lines 7 and 12.

RNA sequencing results were validated technically and biologically with RT-qPCR, as described above. For technical validation, the expression of *StACX3*, *StCS*, *StPti5*, *StPRX28*, and *StTGA2.1* was followed. Biological validation was performed in an independent experiment repetition with TGA2.1-NahG transgenic lines 7 and 12, following gene expression of *StPRX07*, *StPRX15*, *StPRX46*, *StTGA2.1*, and *PVY*. For biological validation, 2–15 early visible lesions and their immediate surroundings were sampled from PVY-inoculated leaves of DEX-treated TGA2.1-NahG, NahG, and NT plants at 4 dpi, as described previously (Lukan et al., 2020). About 20–30 sections of comparable size were sampled from mock-inoculated leaves as controls. Three plants per genotype per treatment were analyzed, pooling together all lesions or mock sections from one leaf per plant. Total RNA was isolated as described previously (Lukan et al., 2020). *StCOX1* and *StEF-1* were used for normalization in both cases, as described above. A two-tailed *t* test was used to compare treatments, when applicable.

All primers and probes used for RT-qPCR analysis together with the target gene IDs are listed in Supplemental Table S12. RT-qPCR assays, targeting *StPRX07*, *StPRX15*, *StPRX46*, *StTGA2.1*, and *StPti5* were designed with Primer Express v2.0 (Applied Biosystems, USA), using the sequences from the potato reference genome (Xu et al., 2011), cultivar Rywal cds, and cultivar Rywal and cultivar Désirée reference transcriptomes (Petek et al., 2020).

### RNA sequencing analysis

For RNA sequencing, 2–25 early visible lesions and their immediate surroundings were sampled from PVY-inoculated leaves of DEX-treated TGA2.1-NahG line 7, NahG and NT plants and control-treated TGA2.1-NahG line 7 plants at 4 dpi, as described previously (Lukan et al., 2020). About 20–30 sections of comparable size were sampled from mock-inoculated leaves as controls. Three plants per genotype per treatment were analyzed, pooling together all lesions or mock sections from one leaf per plant. Total RNA was isolated as described previously (Lukan et al., 2020). Strand-specific library preparation and sequencing were performed by Novogene (HongKong), using the NovaSeq platform (Illumina) to generate 150-bp paired-end reads. Read quality control was performed using FastQC (Andrews, 2010). The presence of contaminant organism reads was determined using Centrifuge (Kim et al., 2016). Reads were mapped to the reference group Phureja DM1-3 potato genome v4.04 (Xu et al., 2011) using the merged PGSC and ITAG genome annotation (Petek et al., 2020) and counted using STAR (Dobin et al., 2013) with default parameters. Differential expression analysis was performed in R using the limma package (Smyth et al., 2018). Raw and normalized read counts as well as a processed data table were deposited at GEO under accession number GSE196078. Genes with Benjamini–Hochberg FDR adjusted *P* values <.05 and |log_2_FC| ≤ −1 were considered statistically significantly differentially expressed. The Venn diagram was drawn, according to results obtained with the Gene List Venn Diagram tool (http://genevenn.sourceforge.net/).

Gene Set Enrichment Analysis (Subramanian et al., 2005) was performed using nonfiltered normalized counts to search for regulated processes and functionally related gene groups, altered significantly by virus inoculation in different genotypes (FDR corrected *q* value <.05) using MapMan ontology (Ramšak et al., 2014) as the source of gene groups.

### Targeted genomic sequencing

Genomic DNA was isolated from potato cultivar Rywal leaves using the DNeasy Plant Mini Kit (Qiagen, Germany). Two sets of primers were designed to target the region of interest (Supplemental Figure S1A, Supplemental Table S13). Droplet-based PCR-free target region enrichment, library preparation using the SQK-LSK109 kit (Oxford Nanopore Technologies, United Kingdom) and long-read sequencing on the MinION platform using the R9.4.1-type flow cell was performed by Samplix (Denmark). Nanopore read basecalling was performed using Guppy 4.2.2. The reads were error corrected with NECAT (Chen et al., 2021) setting GENOME_SIZE = 100,000,000 and PREP_OUTPUT_COVERAGE = 20,000. Chimeric reads were split using Pacasus (Warris et al., 2018) and all reads designated as “passed” were mapped to the group Phureja DM1-3 potato genome v6.1 (Pham et al., 2020) using Minimap2 (Li, 2018). The obtained BAM file was indexed and sorted using SAMtools (Danecek et al., 2021). Raw Nanopore reads were deposited at SRA under accession number PRJNA803339.

### Transactivation assay

GFP-tagged StTGA2.1, BFP-tagged StTGA2.3 and their combination were transiently expressed in *N. benthamiana* leaves, with N7-BFP and either a GFP-tagged SNF-related serine/threonine-protein kinase (StSAPK8) or an empty pH7FWG2 vector (Karimi et al., 2002) as controls. Protein fluorescence was confirmed with confocal microscopy after 3–5 d. The transactivation assays were performed as described previously (Lasierra and Prat, 2018). In brief, 0.5-cm-diameter leaf discs were sampled at 4 dpi and preincubated in MS liquid media with 35 *μ*M D-luciferin substrate for 4 h before analysis. Luminescence was measured in 10 min intervals with Centro LB963 Luminometer (Berthold Technologies, Germany). Leaf discs of 17–18 per construct combination were analyzed. A two-tailed *t* test was used to compare samples. The experiment was repeated twice.

### Protein production, purification, characterization, and antibody preparation

For recombinant production of His_6_-tagged StTGA2.1, *Escherichia coli* BL21(DE3) cells were transformed with the pMCSG7 vector (Eschenfeldt et al., 2009) carrying the *StTGA2.1* cds, grown overnight and subsequently transferred to the liquid autoinduction media (Studier, 2005), where they were incubated for 4 h at 37°C and further 20 h at 20°C to produce the protein. Cells were harvested by centrifugation, lysed and the protein purified by nickel affinity chromatography using the His-Trap HP column coupled with SEC using the HiPrep 26/60 Sephacryl S-200 column (GE Healthcare Life Sciences, UK). The protein was eluted into a buffer containing 30 mM Tris, pH 7.5, and 400 mM NaCl, and used for rabbit polyclonal anti-StTGA2.1 antibody preparation, provided by GenScript (USA).

The protein oligomeric state was determined based on SEC elution volume and Gel Filtration LWM Calibration Kit (standard sizes: conalbumin 75 kDa, ovalbumin 44 kDa, carbonic anhydrase 29 kDa, ribonuclease A 13.7 kDa and aprotinin 6.5 kDa, GE Healthcare, USA). Additionally, the His_6_-tag was removed by His_6_-tagged TEV protease cleavage and a secondary nickel affinity chromatography followed by an additional SEC, as well as an anion-exchange chromatography purification step. Chemical cross-linking was performed after His_6_-tag removal, for which the protein buffer was exchanged to 30 mM Hepes, pH 7.5, 400 mM NaCl using ultrafiltration with Amicon Ultra centrifugal filter units (Merck, Germany). The reaction was performed using the BS^3^ crosslinker according to the manufacturer's instructions (Thermo Scientific, USA) and the protein oligomeric state evaluated by SDS-PAGE.

The *E. coli* cell-free protein synthesis (CFPS) was used for the production of StTGA2.3. All CFPS reactions (total volume 30 or 75 *μ*L) were performed as described previously (Dudley et al., 2021), with 20–24 h incubation at 16, 20, or 25°C. Either the empty pEPQD0KN0025 vector (Addgene plasmid #162283) (Dudley et al., 2021) or water was added to the reagent mixture to prepare a CFPS components reference. Proteins were detected by SDS-PAGE and Western blot, using anti-StTGA2.1 antibodies (diluted 1:4,000, GenScript, USA). Additionally, the protein identity was confirmed with mass spectrometry, performed at the Department of Biochemistry and Molecular and Structural Biology at the Jožef Stefan Institute (Slovenia).

### Surface plasmon resonance

Surface plasmon resonance measurements were performed on Biacore T200 (GE Healthcare, USA) at 25°C at the Infrastructural Centre for Analysis of Molecular Interactions, University of Ljubljana (Slovenia). To prepare the DNA, the PRX07p_1, PRX07p_2, PRX15p_1, and PRX46p complementary primers (Integrated DNA Technologies, Belgium, Supplemental Table S14) were mixed in a 2:3 molar ratio (long:short primers) and annealed by cooling the mixtures from 95°C to 4°C. The resulting DNA fragments carried the selected 20 bp promoter regions with a 15-nucleotide overhang that allowed hybridization with the complementary biotinylated S1 primer (Caveney et al., 2019), immobilized on the streptavidin sensor chip (GE Healthcare, USA). StTGA2.3, either alone or premixed with a His_6_-tagged StTGA2.1, protein–DNA-binding experiments were performed in a running buffer containing 25 mM Tris, pH 7.4, 140 mM NaCl, 1 mM MgCl_2_, and 0.005% (v/v) P20. For His_6_-tagged StTGA2.1 DNA-binding assays, the running buffer contained 180 mM instead of 140 mM NaCl. Flow cell 1 was used as a reference and the DNA fragments were injected across the flow cell 2 at a flow rate of 5 µL/min to immobilize 42–105 response units.

A kinetic titration approach was used to study the interactions between the CFPS-produced StTGA2.3 protein, the CFPS components reference that lacked StTGA2.3 or the His_6_-tagged StTGA2.1 (18.75, 37.5, 75, 150, or 300 nM) and the DNA fragments. The highest concentration of total protein (264 µg/mL) and four sequential 1.5-fold dilutions were used for the CFPS-produced StTGA2.3 and the CFPS components reference. The proteins were injected across DNA at five concentrations, with no dissociation time between protein injections, at a flow rate of 30 µL/min. We used the multi cycle kinetics approach to study the interaction between StTGA2.1 (300 nM) premixed with CFPS-produced StTGA2.3 (total protein concentration of 130 µg/mL) with DNA fragments PRX07p_1, PRX07p_2, or PRX15p_1. The proteins were injected over DNA at a flow rate of 30 µL/min with an association time of 120 s and followed by dissociation for 300 s.

Regeneration of the sensor surface was performed with 50 mM NaOH solution for 10 and 300 mM NaCl for 10 s at a flow rate of 30 µL/min. The sensorgrams for the StTGA2.3 and/or the StTGA2.1 proteins were double subtracted for the response of the reference flow cell 1 and for the response of the CFPS components reference or of the running buffer, respectively.

## Supplementary Material

kiac579_Supplementary_Data

## Accession numbers

Sequence data from this article can be found in the GenBank data library under accession numbers: *StTGA2.1* cds (OM569617), *StTGA2.2* cds (OM569618), *StTGA2.3* cds (OM569619), *StNPR1* cds (OM569620), *StNPR3/4* cds (OM569621), *StPRX07* promoter (OM569622), *StPRX15* promoter (OM569623), and *StPRX46* promoter (OM569624).

## Supplemental data

The following materials are available in the online version of this article.

**Supplemental Figure S1.** Targeted long-read sequencing confirms the presence of mini-TGAs in potato genome.

**Supplemental Figure S2.** PVY replication in the second salicylic acid-deficient transgenic line overexpressing *StTGA2.1*.

**Supplemental Figure S3.** Protein interaction analysis shows the mini-TGA StTGA2.1 can form homodimers *in planta* and *in vitro*.

**Supplemental Figure S4.** Protein interactions between StTGAs and StNPR cofactors in yeast.

**Supplemental Figure S5.** Diverse localization patterns of StTGA2.1.

**Supplemental Figure S6.** StTGA2.2 and StTGA2.3 subnuclear formations.

**Supplemental Figure S7.** RNA sequencing sampling procedure and gene expression analysis Venn diagram.

**Supplemental Figure S8.** Interaction between StTGA2.1 or StTGA2.1 premixed with StTGA2.3 and selected TGA-binding sites and transactivation assay repetition.

**Supplemental Figure S9.** Comparative structural analysis and persistent contacts in dimers of StTGA2.1 and StTGA2.2.

**Supplemental Figure S10.** Probability density of residues of StTGA2.2 forming contacts with a dimer partner.

**Supplemental Table S1.** A list of identified StTGA orthologs including basic protein information.

**Supplemental Table S2.** Differential expression of StTGAs in NT and NahG genotypes after viral infection.

**Supplemental Table S3.** Differential gene expression in salicylic acid-deficient transgenic plants overexpressing *StTGA2.1* after viral infection (available as an Excel file).

**Supplemental Table S4.** Technical validation of RNA sequencing results with RT-qPCR.

**Supplemental Table S5.** Enrichment of differentially regulated genes in salicylic acid-deficient transgenic plants overexpressing *StTGA2.1* after viral infection (available as an Excel file).

**Supplemental Table S6.** Classification of selected potato peroxidases.

**Supplemental Table S7.** Biological validation of RNA sequencing results with RT-qPCR.

**Supplemental Table S8.** Interactions between the StTGA2.2 homodimer and DNA in molecular dynamics simulations.

**Supplemental Table S9.** Interactions between the StTGA2.1 in heterodimer and DNA in molecular dynamics simulations.

**Supplemental Table S10.** Molecular dynamics simulations-based protocol for structure refinement of free and DNA-bound TGA dimers.

**Supplemental Table S11.** Primers used for cloning and sequencing (available as an Excel file).

**Supplemental Table S12.** Primers and probes used for RT-qPCR analysis.

**Supplemental Table S13.** Primers used for targeted genome sequencing.

**Supplemental Table S14.** Complementary primers used for preparation of promoter DNA fragments.
