# Supplementary material for: A mini-TGA protein modulates gene expression through heterogeneous association with transcription factors
Source: Plant Physiol. 2022 Dec 15;191(3):1934–52. doi: 10.1093/plphys/kiac579 (PMC10022624; doi:10.1093/plphys/kiac579)
Supplement: kiac579_Supplementary_Data [file kiac579_supplementary_data.zip › Supplemental_Data.pdf]

Supplemental Data

Title: A mini-TGA protein modulates gene expression through heterogeneous association with transcription factors

Authors: Špela Tomaž*, Marko Petek, Tjaša Lukan, Karmen Pogačar, Katja Stare, Erica Teixeira Prates, Daniel A. Jacobson, Jan Zrimec, Gregor Bajc, Matej Butala, Maruša Pompe Novak, Quentin Dudley, Nicola Patron, Ajda Taler-Verčič, Aleksandra Usenik, Dušan Turk, Salomé Prat, Anna Coll** & Kristina Gruden**

*Corresponding author: Š. Tomaž (spela.tomaz@nib.si), +386 (0)59 232 833.

**Authors contributed equally and share the last authorship.

Supplemental Figures

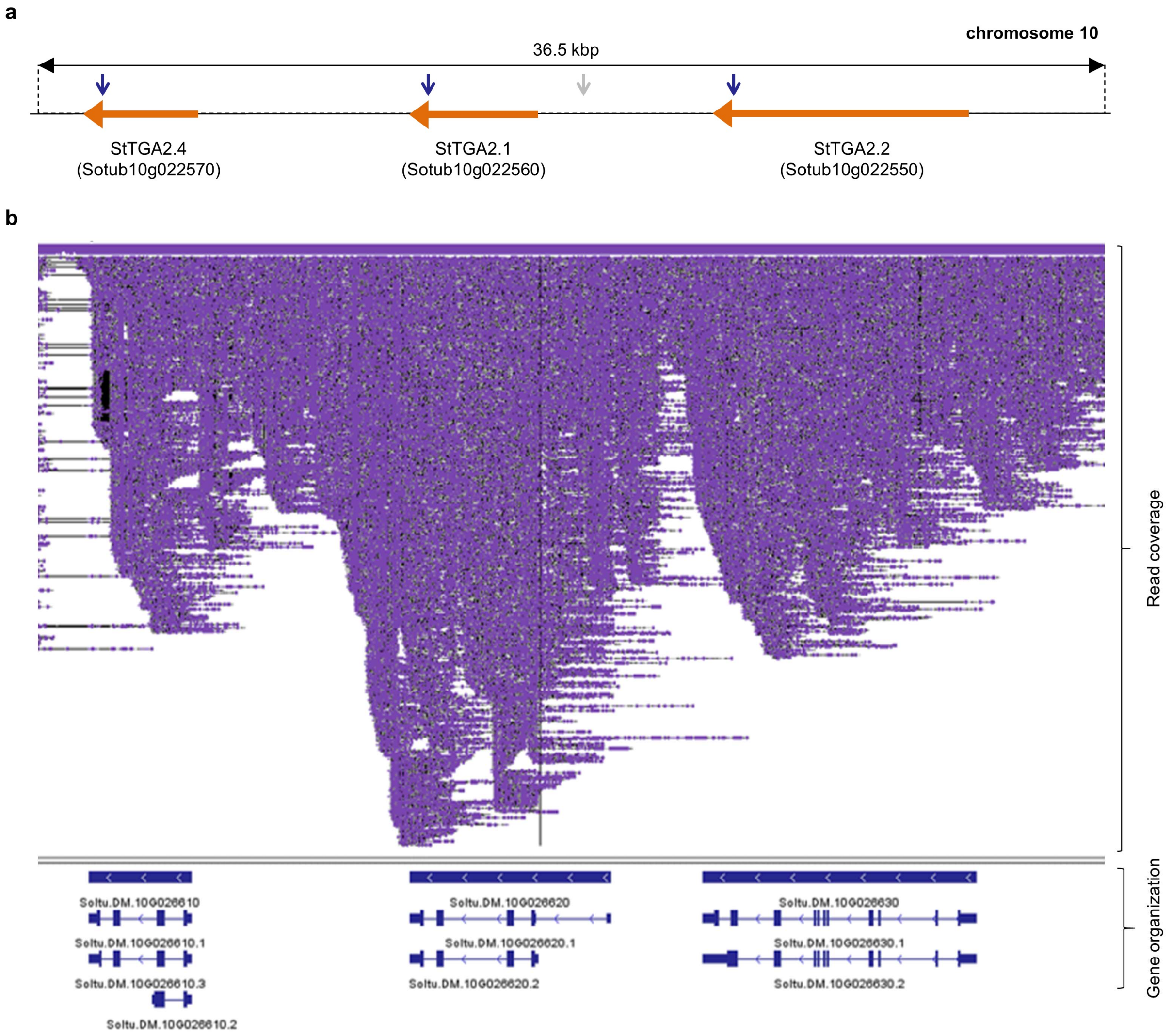

Supplemental Figure S1. Targeted long-read sequencing confirms the presence of mini-TGAs in potato genome. a, Schematic representation of a ~36.5 kbp region on chromosome 10 in the double-monoploid (DM) potato reference genome v6.1 (Pham et al., 2020), encompassing the three tandemly repeated StTGA genes, *StTGA2.1*, *StTGA2.2* and *StTGA2.4* (ROI). All three genes are located on the negative strand. Approximate annealing sites of primer pairs used in targeted long-read sequencing are indicated, with primer pair A (blue arrows) targeting all three genes, and primer pair B (grey arrow) targeting a central part of the ROI. The primers are listed in Supplemental Table S13. **b,** Screenshot, showing the read mapping (purple), obtained through targeted long-read sequencing of the ROI in the tetraploid potato cultivar Rywal, to the potato reference genome v6.1 (Pham et al., 2020). Complete sequence coverage (mean depth 569 at 99.6 % coverage of the ROI) confirms the presence and organization of the three *StTGAs*. Gene organization with several predicted alternative splicing variants, as represented in the DM genome with corresponding transcript IDs, is depicted at the bottom of the figure.

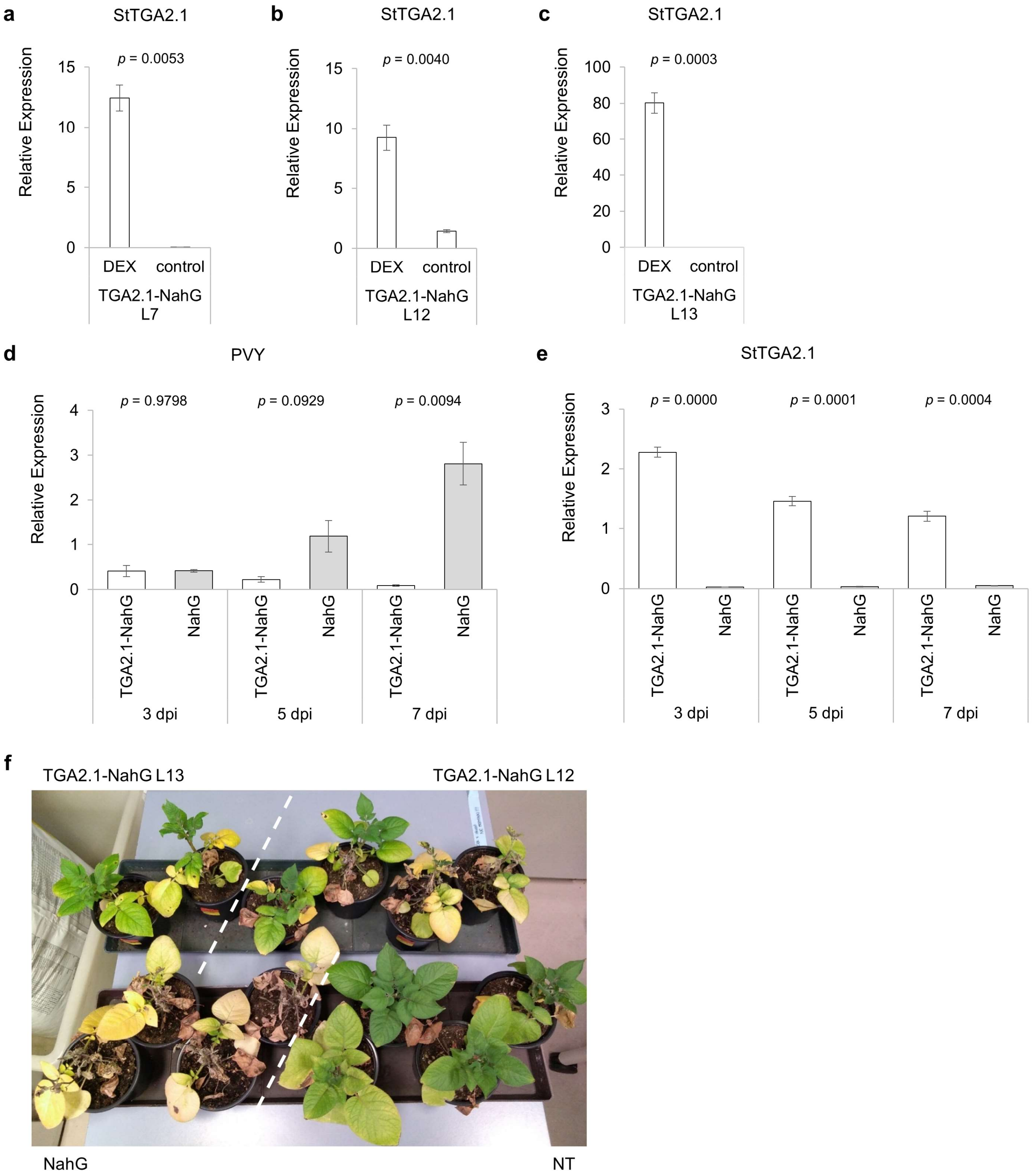

Supplemental Figure S2. PVY replication in the second salicylic acid-deficient transgenic line overexpressing *StTGA2.1*. Relative expression levels of *StTGA2.1* in three NahG transgenic lines overexpressing *StTGA2.1* (TGA2.1-NahG), **a,** line 7 (L7), **b,** line 12 (L12) and **c**, line 13 (L13), three hours after dexamethasone (DEX) treatment, compared to non-treated plants (control). Average values ± standard error from three biological replicates for DEX treatment and **a,** two or **b, c,** three replicates for control are shown. Relative expression levels of **d,** PVY and **e,** *StTGA2.1* in PVY-infected leaves of DEX-treated TGA2.1-NahG L7 (white) and NahG (light grey) plants at 3, 5 and 7 days post infection (dpi). Average values ± standard error from three biological replicates are shown. **a – e,** Significance (p-value < 0.05) was determined using a two-tailed *t*-test. **f,** Phenotypic differences in TGA2.1-NahG L12 and L13, NahG and non-transgenic potato (NT) plants at 32 dpi. Plants from different genotypes are delineated with white dotted lines.

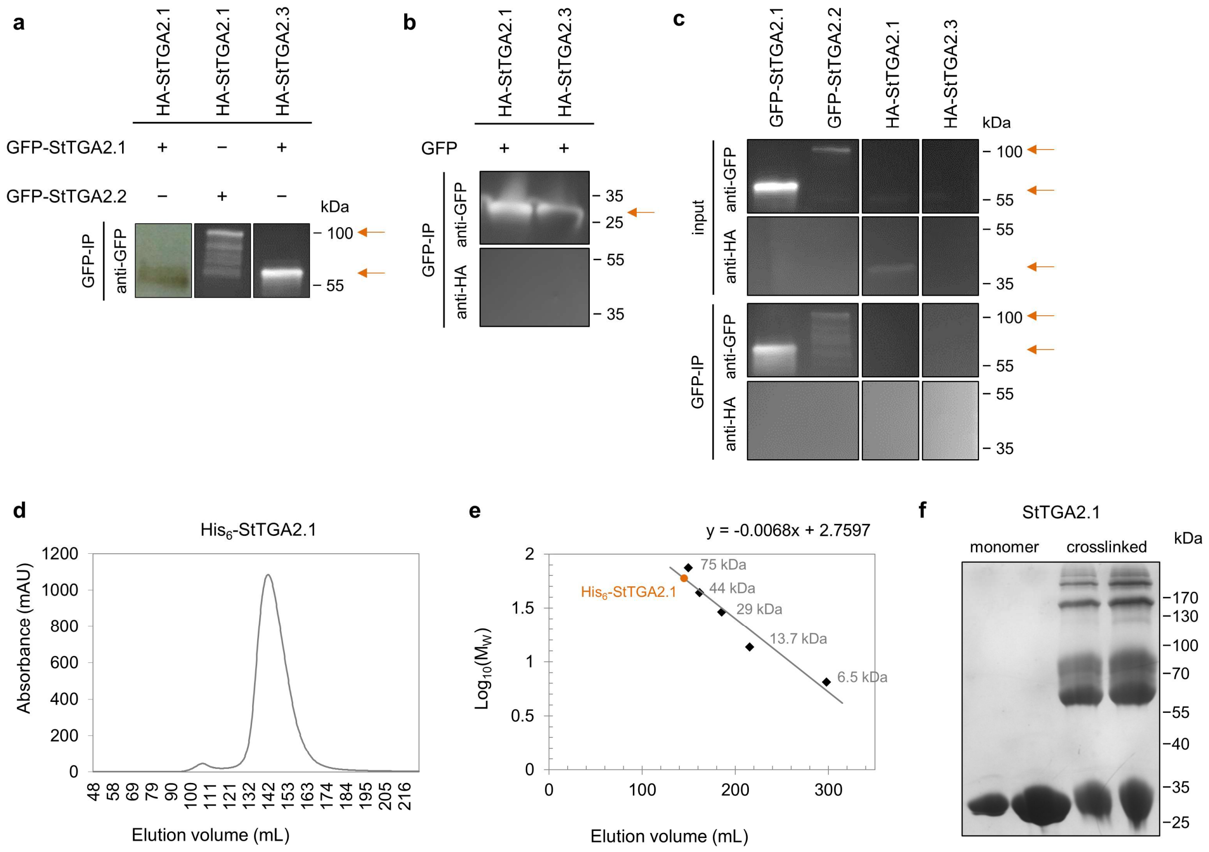

Supplemental Figure S3. Protein interaction analysis shows the mini-TGA StTGA2.1 can form homodimers *in planta* and *in vitro*. a, Co-immunoprecipitation assay positive controls for green fluorescent protein (GFP), showing the detection of GFP-tagged StTGAs after immunoprecipitation (GFP-IP) in the protein-interaction analysis samples (Figure 3b). The combination of GFP and hemagglutinin A (HA)-tagged proteins expressed in *N. benthamiana* is indicated for each sample (+/−). **b,** Negative controls for GFP, showing the HA-tagged proteins do not interact with GFP alone. **c**, Additional controls, showing individual GFP-tagged StTGAs could be detected in the leaf protein extracts (input) and GFP-IP samples. The HA-tagged StTGA2.1 alone was detected in the input, while none of the individual HA-tagged StTGAs were detected in after GFP-IP. **a−c,** Arrows indicate expected bands. **d,** Size exclusion chromatography elution volume of a purified hexahistidine (His_6)-tagged StTGA2.1. **e,** The protein standard calibration curve used to calculate its oligomeric state. The StTGA2.1 elution volume (orange dot) in relation to the protein standard (black diamonds) corresponds to an ~63 kDa large protein, which is twice the size of a monomer (~33 kDa). Protein sizes are depicted next to each standard marker. **f,** StTGA2.1 oligomeric state on SDS-PAGE gel, before (monomer) and after crosslinking (crosslinked), showing the formation of homodimers at ~60 kDa and higher order complexes at ~90, ~160 kDa and above. Two protein amounts per treatment were loaded.

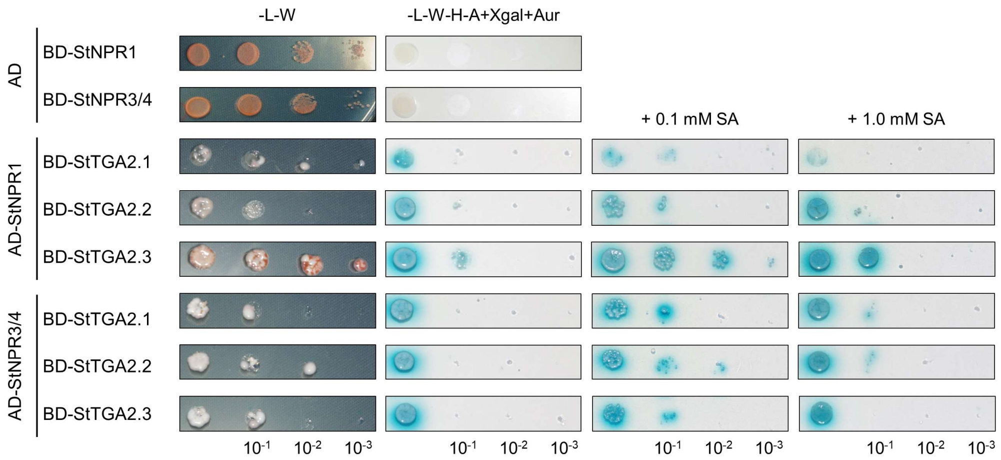

Supplemental Figure S4. Protein interactions between StTGAs and StNPR cofactors in yeast. StTGA2.1, StTGA2.2, and StTGA2.3 interactions with StNPR1 and StNPR3/4 in the yeast two-hybrid assay. Yeast were co-transformed with bait (BD) and prey (AD) construct combinations and selected on control media without Leu and Trp (-L-W). Positive interactions were determined by yeast growth on selection media without Leu, Trp, His and Ade, with added X-α-galactosidase and Aureobasidin A (-L-W-H-A+Xgal+Aur). The effect of salicylic acid (SA) on interaction strength was determined by yeast growth on the same selection media with added 0.1 mM or 1.0 mM SA.

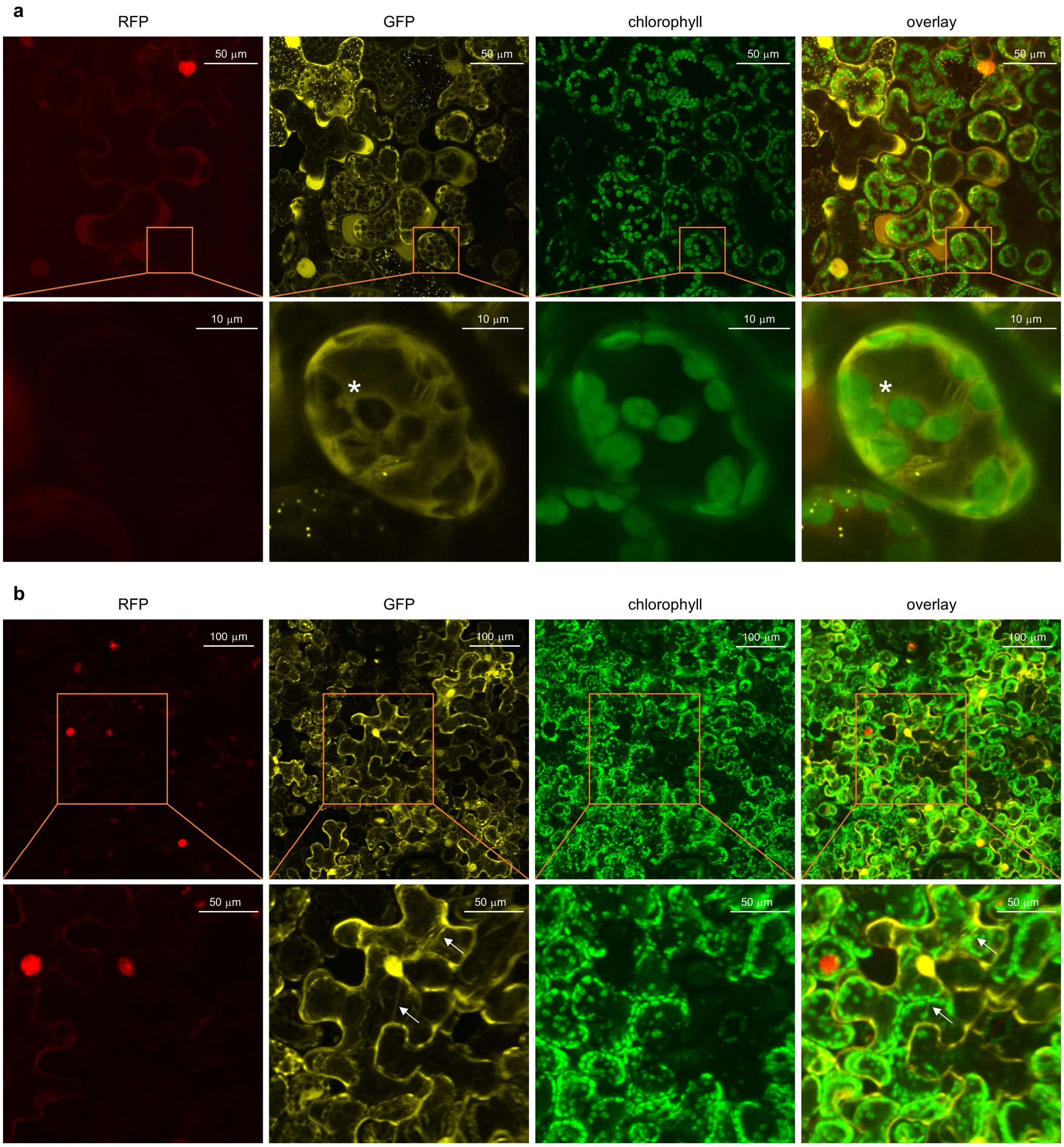

Supplemental Figure S5. Diverse localization patterns of StTGA2.1. Subcellular localization of green fluorescent protein (GFP)-tagged StTGA2.1 (yellow) with Histone 2B-monomeric red fluorescent protein 1 (H2B-RFP) nuclear marker (red) and chloroplast autofluorescence (green) in *N. benthamiana* leaves, showing **a,** StTGA2.1 enrichment around chloroplasts (asterisk) and **b,** localization in the ER (white arrows). **a, b,** Protein fluorescence is represented as the z-stack maximum projection. Orange lines depict the image section close-up. Scale bars, **a,** 50 µm, close-up 10 µm, and **b,** 100 µm, close-up 50 µm.

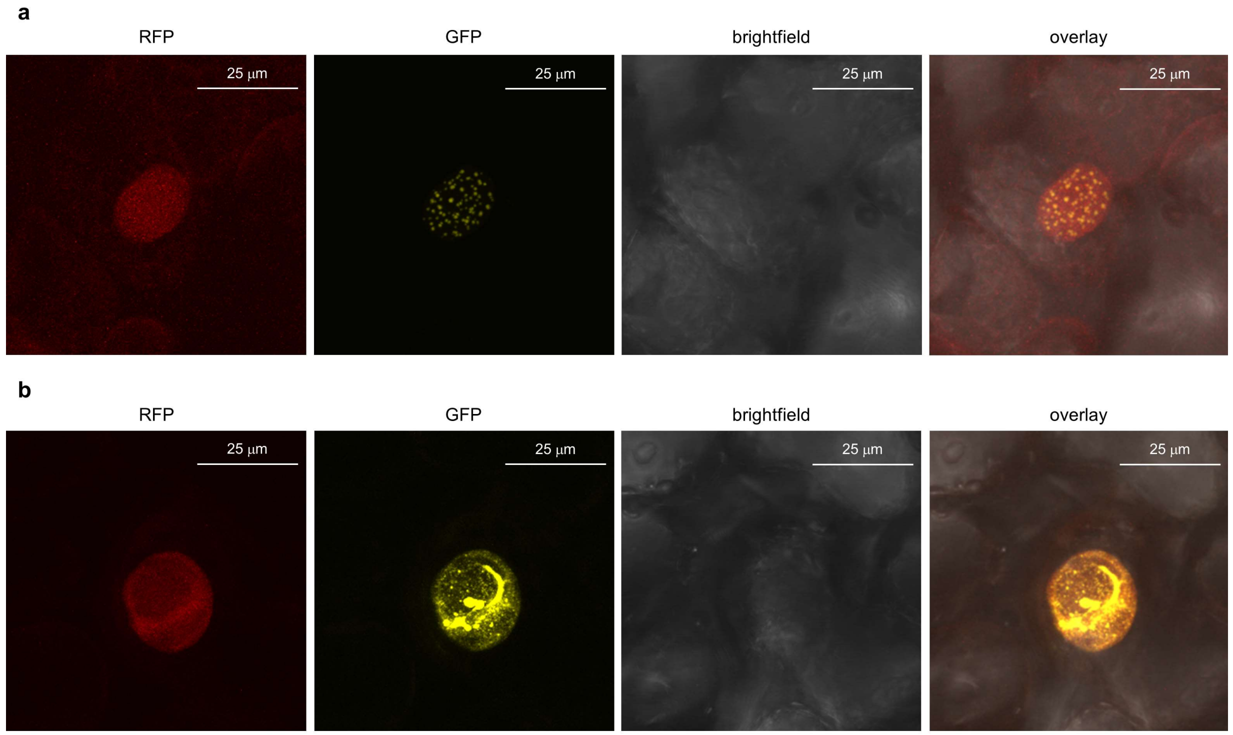

Supplemental Figure S6. StTGA2.2 and StTGA2.3 subnuclear formations. Subnuclear localization of green fluorescent protein (GFP)-tagged **a,** StTGA2.2 and **b,** StTGA2.3 (yellow) with Histone 2B-monomeric red fluorescent protein 1 (H2B-RFP) nuclear marker (red) in *N. benthamiana* leaves. **a, b,** Protein fluorescence is represented as the z-stack maximum projection. Scale bars, 25 μm.

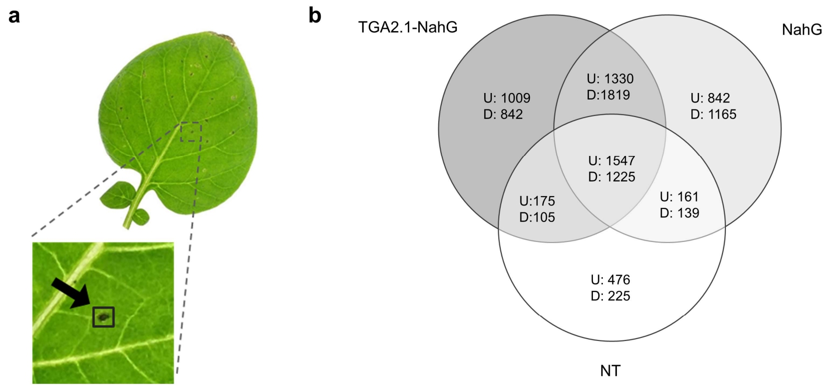

Supplemental Figure S7. RNA sequencing sampling procedure and gene expression analysis Venn diagram. a, Lesions with their immediate surrounding area were cut-out from PVY-inoculated leaves at four days post infection and pooled for RNA sequencing analysis. Mock-inoculated leaf sections of similar size were sampled as controls. **b,** Venn diagram of differentially expressed genes from RNA sequencing between PVY and mock-inoculated TGA2.1-NahG, NahG and non-transgenic (NT) plants. Genes with adjusted p-value < 0.05 and $|\log_2 FC| \leq -1$ were considered significantly differentially expressed. U, up-regulated genes; D, down-regulated genes.

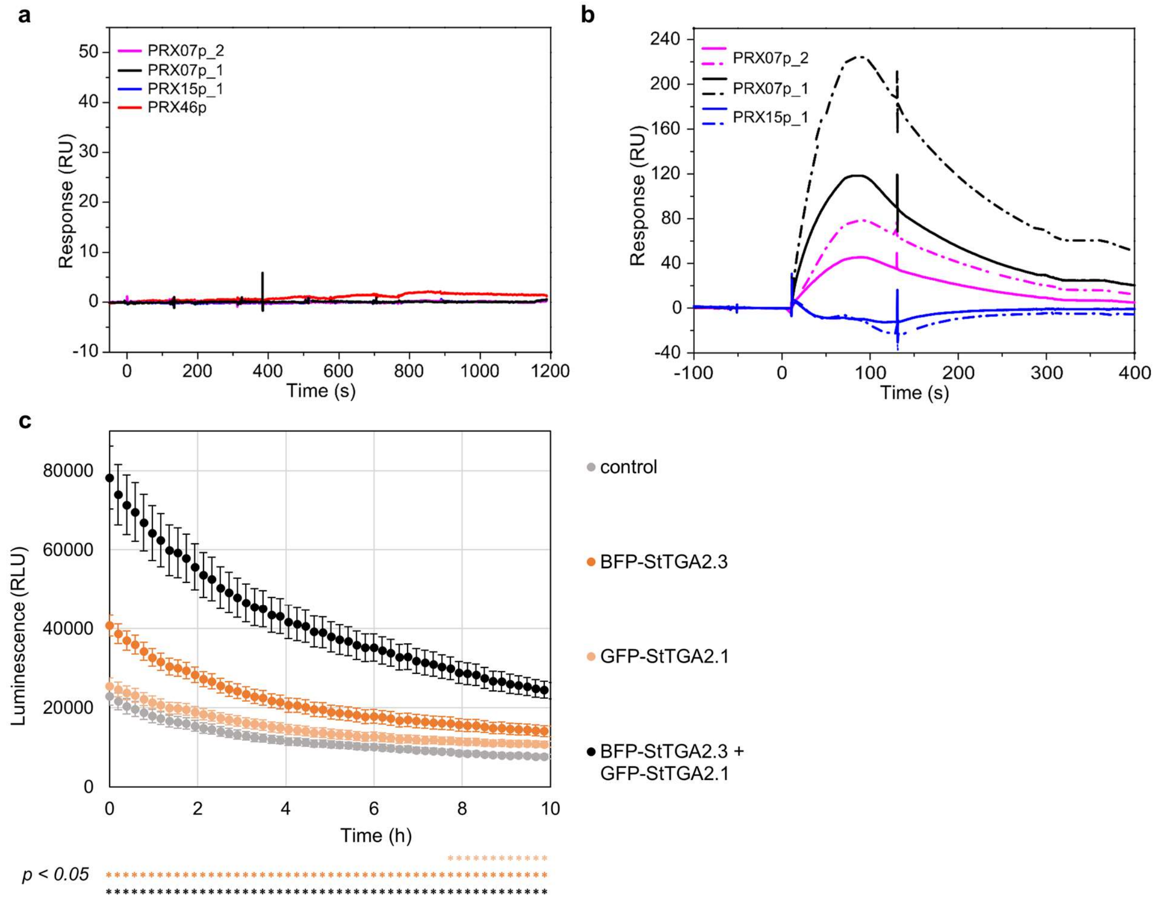

Supplemental Figure S8. Interaction between StTGA2.1 or StTGA2.1 premixed with StTGA2.3 and selected TGA-binding sites and transactivation assay repetition. a, Surface plasmon resonance results, showing no interaction between the hexahistidine (His_6)-tagged StTGA2.1 protein and the chip-immobilized PRX15p_1, PRX46p, PRX07p_1 or PRX07p_2 DNA fragments, bound to the chip at ~77, 42, 55, or 60 response units (RU), respectively. Representative sensorgrams are shown. **b,** Interaction analysis between StTGA2.3 alone (solid line) or premixed with His_6-tagged StTGA2.1 (dashed line) and the chip-immobilized PRX15p_1, PRX07p_1 or PRX07p_2 DNA fragments, bound to the chip at ~105, 105 or 48 RU, respectively. **c,** Transactivation assay repetition, showing *in planta StPRX07* promoter activation by green fluorescent protein (GFP)-tagged StTGA2.1 (light orange), blue fluorescent protein (BFP)-tagged StTGA2.3 (dark orange) or a combination of both (black). BFP or GFP-tagged controls and their combination (control) were used to detect the basal promoter activity (grey). Average values ± standard error of 17 biological replicates in the first 10 h of measurement are shown. Significance (p-value <0.05) was determined using a two-tailed *t*-test and is shown below the response curve for GFP-tagged StTGA2.1 (light orange), BFP-tagged StTGA2.3 (dark orange) or a combination of both (black) compared with control. RLU, relative light units.

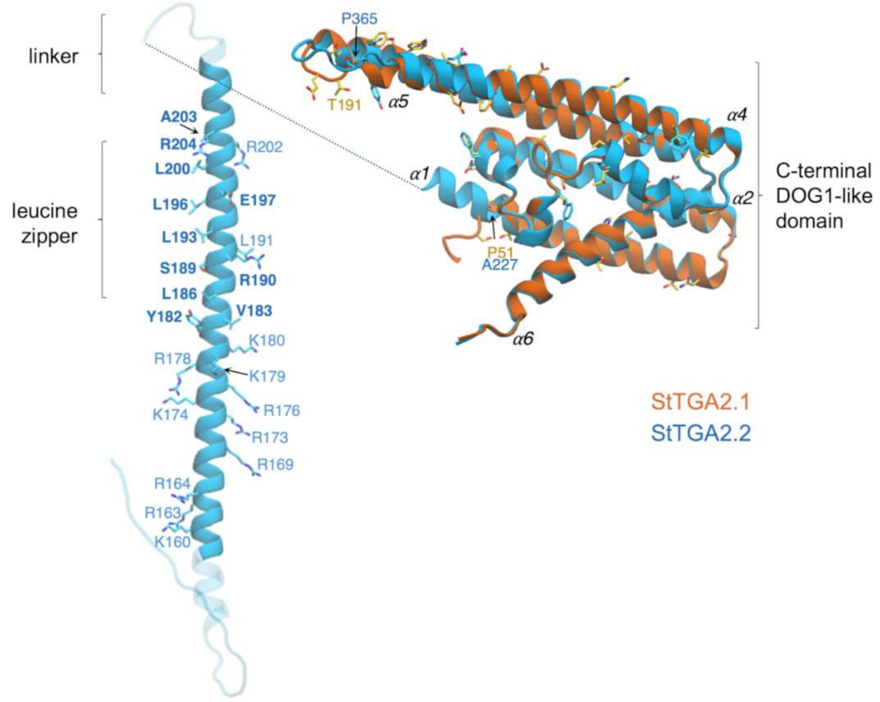

Supplemental Figure S9. Comparative structural analysis and persistent contacts in dimers of StTGA2.1 and StTGA2.2. Molecular architectures of StTGA2.1 (orange) and StTGA2.2 (blue) proteins. The StTGA2.2 bZIP domain (aa 158-211) is shown. The N-termini of StTGA2.1 (aa 1-45) and StTGA2.2 (aa 1-129) are not shown for visual clarity. The C-terminal part is highly conserved between StTGA2.1 (aa 47-240), StTGA2.2 (aa 222-446), and StTGA2.3 (aa 105-327) (Figure 5a). Helices in the C-terminal part are labelled (α1-α6). Amino acid residues forming persistent contacts in the leucine zipper, according to molecular dynamics simulations, are shown in bold. Basic amino acid residues that may contribute to DNA-binding, in the region aa 158-181, are depicted and labelled. Non-conservative substitution sites in the putative DOG1 domains are also represented as liquorice and are labelled for StTGA2.1 (light orange) and StTGA2.2 (blue).

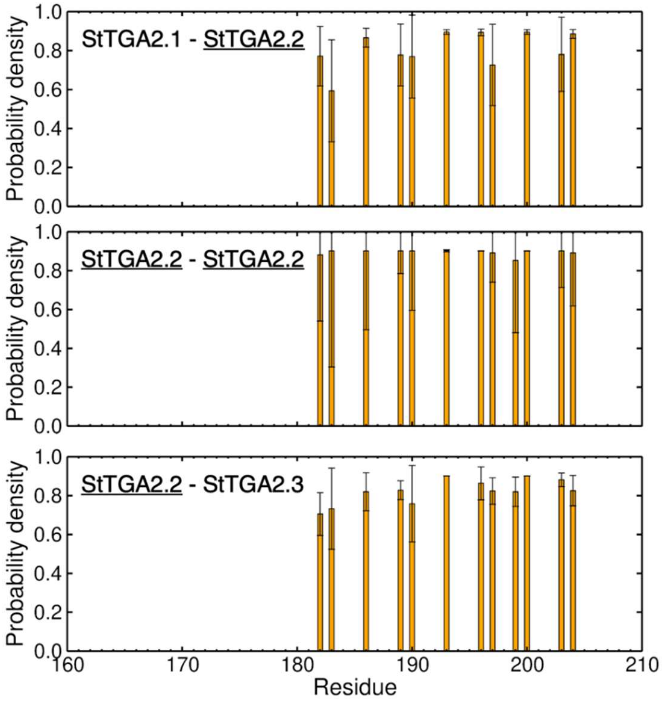

Supplemental Figure S10. Probability density of residues of StTGA2.2 forming contacts with a dimer partner. The truncated protein forming a dimer with StTGA2.2 is specified in each plot (i.e., StTGA2.1, StTGA2.2, or StTGA2.3). A maximum distance of 7 Å between C_α atoms in a pair of residues was established. Bars with a standard deviation > 50% of the probability density are considered transient contacts in the simulations and are not included in these plots. In the StTGA2.2-StTGA2.2 and StTGA2.2-StTGA2.3 dimers the main interacting sites in StTGA2.2 are Tyr182, Val183, Leu186, Ser189, Arg190, Leu193, Leu196, Glu197, Leu200, Gln201, Ala203 and Arg204. The same interacting sites are kept in StTGA2.1-StTGA2.2, except for Glu197. In the simulations, StTGA2.1, StTGA2.2 and StTGA2.3, are truncated, keeping the amino acids 1-43, 159-206, and 42-89, respectively. Values of probability density and the standard deviation were computed from five independent molecular dynamics simulations of 128 ns.

Supplemental Tables

Supplemental Table S1. A list of identified StTGA orthologues including basic protein information. Protein sequence lengths, molecular weight (M_W) and theoretical pI, calculated with the ProtParam tool (Gasteiger et al., 2005), and StTGA domain prediction based on Prosite (de Castro et al., 2006).

No.	Gene ID	Chromosome	Length (aa)	M_W (kDa)	pI	Domain 1	Domain 2
**1**	**Sotub01g009430**	1	327	36.34	8.61	bZIP	DOG1
**2**	**Sotub04g010500**	4	369	41.66	6.20	bZIP	DOG1
**3**	**Sotub04g022350**	4	369	41.57	5.46	bZIP	DOG1
**4**	**Sotub04g027470**	4	361	40.88	6.68	bZIP	DOG1
**5**	**Sotub05g007640**	5	483	53.10	6.51	bZIP	DOG1
**6**	**Sotub06g031310**	6	503	55.94	6.53	bZIP	DOG1
**7**	**Sotub10g020240**	10	488	55.53	6.26	bZIP	DOG1
**8**	**Sotub10g022140**	10	461	52.24	6.47	bZIP	DOG1
**9**	**Sotub10g022550**	10	438	48.23	7.18	bZIP	DOG1
**10**	**Sotub10g022560**	10	270	30.49	5.69	-	DOG1
**11**	**Sotub10g022570**	10	271	30.51	6.00	-	DOG1
**12**	**Sotub11g020650**	11	324	36.17	8.86	bZIP	DOG1
**13**	**Sotub11g025820**	11	435	48.60	6.91	bZIP	DOG1
**14**	**Sotub12g025350**	12	348	39.56	6.92	bZIP	DOG1

Supplemental Table S2. Differential expression of StTGAs in NT and NahG genotypes after viral infection. Microarray data showing StTGA gene expression comparisons between PVY- and mock-inoculated plants at 1, 3 and 6 days post inoculation (dpi), adapted from Baebler *et al.* (2014). Only statistically significant values (FDR adjusted p-value < 0.05) are shown, given as log_2FC. Cell shading based on log_2FC values: blue, down-regulated; orange, up-regulated.

		NT			NahG		
**Gene ID**	**Microarray ID**	**1 dpi**	**3 dpi**	**6 dpi**	**1 dpi**	**3 dpi**	**6 dpi**
**Sotub04g010500**	MICRO.14906.C1	-	-	-	1.04	-	-
	MICRO.14906.C2	-	-	-	0.77	-	-0.79
	MICRO.14906.C3	-	-0.93	-0.68	0.49	-0.90	-1.35
	POAED18TP	-	-0.45	-	-	-	-
**Sotub04g022350**	MICRO.7564.C1	0.96	-	-	-	-	-
**Sotub01g009430**	MICRO.14316.C1	-	-	-	-	-	-
	MICRO.14867.C1	0.34	0.68	0.42	0.54	-	0.28
	MICRO.8878.C1	-0.62	-	-	-	-	0.53
	POAC439TP	-0.64	-0.39	-	-	-	0.60
**Sotub10g022550**	MICRO.7441.C1	-1.56	-1.03	0.82	-0.80	1.05	2.63
	MICRO.7474.C3	-0.95	-1.06	-	-	-	-
**Sotub10g022560**	bf_arrayxxx_0075b06.t7m.scf	-	-	-	-	2.11	1.98
	MICRO.7474.C2	-0.41	-	-	0.34	-	0.80
	MICRO.7474.C5	-	-	-	-	0.44	0.87
	STMHL35TV	-0.90	-	-	-	0.70	1.72
**Sotub10g022570**	MICRO.7474.C6	1.14	1.23	-	0.68	-	-
**Sotub04g027470**	bf_arrayxxx_0086h05.t7m.scf	0.82	-	-	-	-	-
	MICRO.11212.C1	-	-	-0.41	-	-0.46	-0.66
	MICRO.9918.C1	-0.37	-0.46	-	-	-0.48	-0.47
	STMGM64TH	-0.91	-1.53	-	-1.00	-	0.44
**Sotub06g031310**	bf_suspxxxx_0045g08.t7m.scf	-	-	-	-	-	0.71
**Sotub10g020240**	bf_ivrootxx_0054a07.t3m.scf	0.49	-0.59	-	-	-	-
**Sotub11g025820**	MICRO.14778.C1	0.67	0.78	1.15	0.71	1.23	2.22
	MICRO.3439.C1	-0.99	-1.29	-0.57	-0.99	-0.41	0.35
**Sotub05g007640**	MICRO.15058.C1	-	-	-	-	-	-0.74
	POAD811TP	-	-	-	-	-	-
	POAD811TV	-	-	-	-	-	-

Supplemental Table S3. Differential gene expression in salicylic acid-deficient transgenic plants overexpressing *StTGA2.1* after viral infection (available as an Excel file). RNA sequencing results table showing gene expression comparisons between dexamethasone (DEX)-treated mock-inoculated plants (TGA2.1-NahG vs. NahG and TGA2.1-NahG vs. NT) and comparisons of DEX-treated PVY- vs mock-inoculated plants of all three genotypes, given as $\log_2$FC and adjusted p-value. Additionally, comparisons between DEX- and control-treatments (DEX vs. control) are shown for PVY and mock-inoculated TGA2.1-NahG plants, as controls. For each gene, the ITAG or PGSC identifier, description and a MapMan ontology functional gene group (BIN) (Ramšak et al., 2014) with BIN number, is listed. Only the first BIN mapping is shown. FDR adjusted p-values below 0.05 are depicted in red. TMM normalized reads counts and raw read counts for each of the three biological replicates per genotype per treatment are shown. Cell shading based on $\log_2$FC values: blue, down-regulated; orange, up-regulated.

Supplemental Table S4. Technical validation of RNA sequencing results with RT-qPCR. Comparison of five differentially expressed genes obtained from RNA sequencing results (RNA-Seq) with RT-qPCR analysis for TGA2.1-NahG, NahG and NT plants 4 days after viral infection (PVY- vs. mock-inoculated plants comparison), given as $\log_2$FC. Gene expression ratios with FDR adjusted p-value < 0.05 are underlined. Cell shading based on $\log_2$FC values: blue, down-regulated; orange, up-regulated.

		TGA2.1-NahG		NahG		NT	
**Gene Name**	**Gene ID**	**RNA-Seq**	**RT-qPCR**	**RNA-Seq**	**RT-qPCR**	**RNA-Seq**	**RT-qPCR**
**StACX3**	Sotub10g008540	2.05	2.07	2.21	2.11	1.46	1.50
**StCS**	Sotub01g027350	1.55	1.17	1.49	1.80	0.52	0.67
**StPti5**	Sotub02g020180	4.37	3.85	5.36	8.25	2.14	5.33
**StPRX28**	Sotub01g042120<br>Sotub02g011690	7.47	6.29	6.91	5.69	2.89	2.48
**StTGA2.1**	Sotub10g022560	-1.45	-1.54	0.21	0.95	-0.26	-0.37

Supplemental Table S5. Enrichment of differentially regulated genes in salicylic acid-deficient transgenic plants overexpressing *StTGA2.1* after viral infection (available as an Excel file). Gene Set Enrichment Analysis results table showing MapMan ontology functional gene groups (BINs) (Ramšak et al., 2014) enriched in up-regulated or down-regulated genes in TGA2.1-NahG, NahG or NT plants after PVY infection. Functional groups, significantly enriched (FDR corrected q-value < 0.05) in at least one of the three genotypes, are listed. (+), enriched in up-regulated genes; (−), enriched in down-regulated genes.

Supplemental Table S6. Classification of selected potato peroxidases. Genes from the MapMan peroxidase functional group (BIN 26.12) (Ramšak et al., 2014) were named and classified according to BLAST results in the RedoxiBase database (Savelli et al., 2019). All hits with E-value equal to zero or best hits with E-value above zero (*) are listed for each gene. The presence of secretory signal peptides was predicted with SignalP 5.0 (Almagro Armenteros et al., 2019). (+), signal peptide; (−), no signal peptide predicted; (+/−) signal peptide predicted in at least one of several sequences assigned to the same gene ID; bold, the three peroxidases included in target confirmation.

Gene ID	BLAST Hits	Classification	Secretory signal Peptide
PGSC0003DMG400020437	StPRX05, StPRX52, StPRX72	class III peroxidase	−
PGSC0003DMG400025492	StPRX72, StPRX52	class III peroxidase	+
PGSC0003DMG400025621	StPRX20, StPRX09, StPRX28, StPRX50	class III peroxidase	−
PGSC0003DMG400026575	StPRX19, StPRX02, StPRX15	class III peroxidase	+/−
PGSC0003DMG400032147	StPRX12, StPRX10	class III peroxidase	+
Sotub01g006690	StPRX20, StPRX09	class III peroxidase	+
Sotub01g042120	StPRX28, StPRX50	class III peroxidase	+
Sotub02g022480	StPRX72, StPRX52	class III peroxidase	+
Sotub02g022490	StPRX72, StPRX52	class III peroxidase	+
Sotub02g023700	StPRX05	class III peroxidase	+
Sotub02g027910	StPRX12, StPRX10	class III peroxidase	−
Sotub02g031070	StPRX19, StPRX02	class III peroxidase	+
**Sotub02g035680**	**StPRX15**	**class III peroxidase**	**+**
Sotub02g037370	StPRX19*	class III peroxidase	+
**Sotub03g007840**	**StPRX46**	**class III peroxidase**	**+**
Sotub03g010400	StPRX19, StPRX02	class III peroxidase	+
Sotub03g015450	StPRX24	class III peroxidase	+
Sotub04g026530	StPRX14	class III peroxidase	+
Sotub04g026540	StPRX11, StPRX17	class III peroxidase	+
Sotub04g026550	StPRX11, StPRX17	class III peroxidase	+
Sotub05g006810	StPRX75	class III peroxidase	−
Sotub05g024920	StPRX47, StPRX16	class III peroxidase	+
Sotub06g009790	StPRX47, StPRX16	class III peroxidase	+
Sotub06g032420	StPRX71	class III peroxidase	+
Sotub06g033210	StPRX25	class III peroxidase	+
Sotub08g006050	StPRX27	class III peroxidase	+
Sotub08g011590	StAPx-R	ascorbate peroxidase related	−
Sotub08g017100	StPRX40	class III peroxidase	+
Sotub09g007100	StPRX03, StPRX04	class III peroxidase	+
**Sotub09g020950**	**StPRX07**	**class III peroxidase**	**+**
Sotub10g024760	StPRX03	class III peroxidase	+
Sotub11g009570	StPRX58	class III peroxidase	+
Sotub11g029720	StPRX51*	class III peroxidase	+

Supplemental Table S7. Biological validation of RNA sequencing results with RT-qPCR. Relative expression of three class III peroxidase genes as obtained by RNA sequencing (RNA-Seq) and RT-qPCR analyses for both TGA2.1-NahG lines, line 7 (L7) and line 12 (L12), NahG and NT plants 4 days after viral infection (PVY- vs. mock-inoculated plants comparison), given as log_2FC. Gene expression ratios with FDR adjusted p-value < 0.05 are underlined. Cell shading based on log_2FC values: blue, down-regulated; orange, up-regulated.

		TGA2.1-NahG			NahG		NT	
**Gene Name**	**Gene ID**	**RNA-Seq**	**RT-qPCR L7**	**RT-qPCR L12**	**RNA-Seq**	**RT-qPCR**	**RNA-Seq**	**RT-qPCR**
**StPRX07**	Sotub09g020950	<u>10.78</u>	<u>6.87</u>	<u>3.86</u>	<u>7.13</u>	3.54	5.19	<u>3.43</u>
**StPRX15**	Sotub02g035680	<u>1.90</u>	1.14	<u>1.59</u>	-0.15	0.20	<u>4.27</u>	<u>3.42</u>
**StPRX46**	Sotub03g007840	<u>6.79</u>	<u>3.92</u>	<u>3.24</u>	<u>2.88</u>	<u>2.21</u>	<u>4.23</u>	<u>1.84</u>
**StTGA2.1**	Sotub10g022560	<u>-1.45</u>	<u>-3.67</u>	0.10	0.21	-0.07	-0.26	-0.50

Supplemental Table S8. Interactions between the StTGA2.2 homodimer and DNA in molecular dynamics simulations. Total frequency of hydrogen bond, salt bridge interactions, and hydrophobic contacts (*) between the protomers of the StTGA2.2 homodimer and the two DNA strands (DNA1 and DNA2) in five independent molecular dynamics simulations of 200 ns. For hydrophobic contacts, a maximum distance of 4 Å between C_β atoms in the protein and the methyl group in a thymine was established.

StTGA2.2	DNA1	Frequency / %					StTGA2.2	DNA2	Frequency / %				
K160	A-8	19	6	8	-	25	K160	-	-	-	-	-	-
R163	T-7	90	-	82	63	59	R163	T-7	33	34	-	20	41
R163	A3	-	28	20	-	-	R163	-	-	-	-	-	-
R164	A-8	27	28	1	19	-	R164	T-7	36	18	19	10	1
Q167	T-7	11	-	22	20	17	Q167	T-6	11	5	14	8	10
N168	A3	4	15	9	7	7	N168	C3	30	29	19	22	16
N168	T-4	26	16	24	23	26	-	-	-	-	-	-	-
R169	T2	22	22	30	14	19	R169	T2	19	13	20	24	24
A171*	T-4	18	20	38	21	24	A171*	T-5	21	34	38	33	44
A172*	T2	43	24	21	21	43	A172*	T2	37	38	36	32	34
R173	C-1	22	65	17	32	24	R173	G1	39	9	9	12	20
R173	G1	12	35	16	17	12	R173	C-1	-	6	7	11	12
S175	T-4	78	80	54	59	63	S175	C-4	60	59	58	49	57
R176	G1	26	38	31	41	39	R176	G1	13	34	20	17	13
R176	-	-	-	-	-	-	R176	C-1	22	24	22	24	27
R178	T-4	37	29	33	33	22	R178	C-4	59	45	26	41	35
R178	G-5	18	-	58	17	24	R178	T-5	13	14	28	24	22
K179	G-3	18	7	31	17	10	K179	T-3	30	29	33	35	38
K180	A-2	22	38	30	28	31	K180	A-2	37	48	30	33	37

Supplemental Table S9. Interactions between the StTGA2.1 in heterodimer and DNA in molecular dynamics simulations. Total frequency of hydrogen bond, salt bridge interactions, and hydrophobic contacts (*) between StTGA2.1 in the StTGA2.1-StTGA2.2 heterodimer and DNA in five independent molecular dynamics simulations of 200 ns. For hydrophobic contacts, a maximum distance of 4 Å between C_β atoms in the protein and the methyl group in a thymine was established.

StTGA2.1	DNA	Frequency / %				
R7	G1	-	10	-	22	-
R7	T2	22	13	22	17	25
R11	G1	31	12	20	27	12
R11	C-1	13	15	39	13	12
R20	T-5	27	5	22	15	0
R20	C-4	28	24	33	37	32
R24	T-3	28	22	15	24	22
A13*	T-3	12	32	6	6	27

Supplemental Table S10. Molecular dynamics simulations-based protocol for structure refinement of free and DNA-bound TGA dimers. In the equilibration phases, there is a gradual change in temperature, time step, and the position restraint potentials. Atom velocities are re-distributed using five different seed numbers to initiate Equilibration_6. With that, five independent trajectories are generated for conformational sampling. Applied potentials are either simple harmonic restraints (H) or flat-bottom potentials (FB). During and after Equilibration_5, the position restraint potentials are applied only to the residues forming the leucine heptads. "Backbone" refers to both the backbone atoms of TGA proteins and DNA.

Simulation Phases	Position Restraints				Time Step	Total Time	Temperature
	Backbone	Type	Side Chain	Type			
**Equilibration_1**	1.000	H	0.100	H	1 fs	2 ns	100.15 K
**Equilibration_2**	1.000	H	0.050	H	2 fs	1 ns	200.15 K
**Equilibration_3**	1.000	H	0.025	H	2 fs	1 ns	250.15 K
**Equilibration_4**	1.000	H	0.000	H	2 fs	1 ns	300.15 K
**Equilibration_5**	0.500*	H	0.000	-	2 fs	4 ns	320.15 K
**Equilibration_6**	0.250*	H	0.000	-	2 fs	5 x 2 ns	340.15 K
**Equilibration_7**	0.125*	H	0.000	-	4 fs	5 x 12 ns	360.15 K
**Equilibration_8**	0.050*	H	0.000	-	4 fs	5 x 128 ns	340.15 K
**Equilibration_9**	0.025*	H	0.000	-	4 fs	5 x 64 ns	320.15 K
**Equilibration_10**	0.250*	FB	0.000	-	4 fs	5 x 100 ns	298.15 K
**Sampling**	0.000	-	0.000	-	4 fs	5 x 128 ns	298.15 K

*Position restraints applied to Ca only.

Supplemental Table S11. Primers used for cloning and sequencing (available as an Excel file).

Supplemental Table S12. Primers and probes used for RT-qPCR analysis. F, forward; R, reverse; P, probe.

Gene Name	Gene Description	Gene ID	Primer/Probe Sequence (5'-3')	Amplicon Efficiency
**StACX3**	acyl-CoA oxidase	Sotub10g008540	Baebler *et al.* (2014)	
**StCS**	citrate synthase	Sotub01g027350		
**StPRX28***	class III peroxidase	Sotub01g042120, Sotub02g011690		
**StPti5**	ethylene responsive factor	Sotub02g020180	F: GCAAGAAATACAGAGGCGTACGA<br>R: CATACTCTCGCACCGTGTCTAG<br>P: FAM-TTCGGCAGCGTATTTT-NFQ	93%
**StTGA2.1**	TGA transcription factor	Sotub10g022560	F: ATCTCTTGCTACTGAAGGGTCAT<br>R: CCATAGCCATTGCCATCTGACTTAT<br>P: FAM-CCGGAGATGCAGCTTATAAT-NFQ	90%
**StPRX07**	class III peroxidase	Sotub09g020950 (CLCdnDe14_116472, CLCdnRY2_25372, CLCdnRY3_29096, VdnDe5_126154)**	F: GTTGGAATCGCACATTGTAATTTT<br>R: AGCTGCTAATGTAGGATCCATAGTCTT<br>P: FAM-TCCAAGATAGGCTATCGCCCGTACCTG-Zen Iowa Black TM FQ	92%
**StPRX15**	class III peroxidase	Sotub02g035680 (CLCdnDe10_14846, CLCdnRY1_7366, CLCdnRY10_8871, VdnDe1_213633, VdnDe1_213635, VdnDe6_46588)**	F: CACTACAAACAAACAGATCACTTCACCTA<br>R: GACGAAGCAGTCGTGGAAGAA<br>P: FAM-TACCGCCGCCGCCACCCT-Zen Iowa Black TM FQ	80%
**StPRX46**	class III peroxidase	Sotub03g007840 (CLCdnDe11_47734, CLCdnRY1_4154, PBdnRY1_4594, VdnDe1_299904, VdnDe1_299906, VdnDe6_44388)**	F: CGGCGGTTGGGTCAGTT<br>R: GAATGAGAGATGCTCCCATTCG<br>P: FAM-TGGCGGAAGCTATTGCGAGGGA-Zen Iowa Black TM FQ	91%
**StCOX1**	cytochrome oxidase	Sotub04g015050	Weller *et al.* (2000)	
**StEF-1**	elongation factor	Sotub06g010680	Baebler *et al.* (2009)	
**PVY Univ**	PVY coat protein	AJ390300	Kogovšek *et al.* (2008)	

* Previously named POX (Baebler et al., 2014).

** For RT-qPCR assay design we used sequences obtained from cultivar Rywal and cultivar Désirée reference transcriptome(Petek et al., 2020) as template. Only transcripts, targeted by the assay, are listed.

Supplemental Table S13. Primers used for targeted genome sequencing.

Primer Pair	Primer Name	Sequence (5’-3’)
A	WO100007-NIB-1_7F	GAGGCAAAATACGTGAAGCTAAAAG
	WO100007-NIB-1_7R	AGAGCACGGGCTGATTGTCT
B	WO100007-1_3F	GCTACGCCTTTGCAGCATAT
	WO100007-1_3R	ACAGGAAAAGCAACTCTGGCT

Supplemental Table S14. Complementary primers used for preparation of promoter DNA fragments.

Gene Name and ID	Promoter Fragment	Primer Name	Sequence (5'-3')
**StPRX07** Sotub09g020950	PRX07p_1	per950p_1F	GTTACTACTCGAGCGTGTGCCCAACGTCACAATCC
		per950p_1R	GGATTGTGACGTTGGGCACA
	PRX07p_2	per950p_2F	GTTACTACTCGAGCGTTAGGGGTGACGTTTCCAAT
		per950p_2R	ATTGGAAACGTCACCCCTAA
**StPRX15** Sotub02g035680	PRX15p_1	per680p_1F	GTTACTACTCGAGCGTTTAATAATGATGACATTTG
		per680p_1R	CAAATGTCATCATTATTAAA
**StPRX46** Sotub03g007840	PRX46p	per840p_1F	GTTACTACTCGAGCGTGAACCTGAGGTCAACCGTT
		per840p_1R	AACGGTTGACCTCAGGTTCA

Supplemental References

Almagro Armenteros JJ, Tsirigos KD, Sønderby CK, Petersen TN, Winther O, Brunak S, von Heijne G, Nielsen H (2019) SignalP 5.0 improves signal peptide predictions using deep neural networks. Nat Biotechnol **37**: 420–423

Aoyama T, Chua NH (1997) A glucocorticoid-mediated transcriptional induction system in transgenic plants. Plant J **11**: 605–612

Baebler Š, Krečič-Stres H, Rotter A, Kogovšek P, Cankar K, Kok EJ, Gruden K, Kovač M, Žel J, Pompe-Novak M, et al (2009) PVYNTN elicits a diverse gene expression response in different potato genotypes in the first 12 h after inoculation. Mol Plant Pathol **10**: 263–275

Baebler Š, Witek K, Petek M, Stare K, Tušek-Žnidarič M, Pompe-Novak M, Renaut J, Szajko K, Strzelczyk-Zyta D, Marczewski W, et al (2014) Salicylic acid is an indispensable component of the Ny-1 resistance-gene-mediated response against Potato virus Y infection in potato. J Exp Bot **65**: 1095–1109

de Castro E, Sigrist CJA, Gattiker A, Bulliard V, Langendijk-Genevaux PS, Gasteiger E, Bairoch A, Hulo N (2006) ScanProsite: Detection of PROSITE signature matches and ProRule-associated functional and structural residues in proteins. Nucleic Acids Res **34**: 362–365

Dudley QM, Cai YM, Kallam K, Debreyne H, Carrasco Lopez JA, Patron NJ (2021) Biofoundry-assisted expression and characterization of plant proteins. Synth Biol **6**: ysab029

Eschenfeldt WH, Stols L, Sanville Millard C, Joachimiak A, Donnelly MI (2009) A Family of LIC Vectors for High-Throughput Cloning and Purification of Proteins. Methods Mol Biol **498**: 105–115

Gasteiger E, Hoogland C, Gattiker A, Duvaud S, Wilkins MR, Appel RD, Bairoch A (2005) Protein identification and analysis tools on the ExPASy server. *In* JM Walker, ed, Proteomics Protoc. Handb., 1st ed. Humana Press, pp 571–607

Kogovšek P, Gow L, Pompe-Novak M, Gruden K, Foster GD, Boonham N, Ravnikar M (2008) Single-step RT real-time PCR for sensitive detection and discrimination of Potato virus Y isolates. J Virol Methods **149**: 1–11

Petek M, Zagorščak M, Ramšak Ž, Sanders S, Tomaž Š, Tseng E, Zouine M, Coll A, Gruden K (2020) Cultivar-specific transcriptome and pan-transcriptome reconstruction of tetraploid potato. Sci Data **7**: 249

Pham GM, Hamilton JP, Wood JC, Burke JT, Zhao H, Vaillancourt B, Ou S, Jiang J, Robin Buell C (2020) Construction of a chromosome-scale long-read reference genome assembly for potato. Gigascience **9**: giaa100

Ramšak Ž, Baebler Š, Rotter A, Korbar M, Mozetič I, Usadel B, Gruden K (2014) GoMapMan: integration, consolidation and visualization of plant gene annotations within the MapMan ontology. Nucleic Acids Res **42**: 1167–1175

Savelli B, Li Q, Webber M, Jemmat AM, Robitaille A, Zamocky M, Mathé C, Dunand C (2019) RedoxiBase: A database for ROS homeostasis regulated proteins. Redox Biol **26**: 101247

Stark JC, Huang A, Nguyen PQ, Dubner RS, Hsu KJ, Ferrante TC, Anderson M, Kanapskyte A, Mucha Q, Packett JS, et al (2018) BioBits^{TM} Bright: A fluorescent synthetic biology education kit. Sci Adv **4**: eaat5107

Weller SA, Elphinstone JG, Smith NC, Boonham N, Stead DE (2000) Detection of Ralstonia solanacearum strains with a quantitative, multiplex, real-time, fluorogenic PCR (TaqMan) assay. Appl Environ Microbiol **66**: 2853–2858
